# Vascular dysfunction and increased cardiovascular risk in hypospadias

**DOI:** 10.1093/eurheartj/ehac112

**Published:** 2022-03-17

**Authors:** Angela K Lucas-Herald, Augusto C Montezano, Rheure Alves-Lopes, Laura Haddow, Malika Alimussina, Stuart O’Toole, Martyn Flett, Boma Lee, S Basith Amjad, Mairi Steven, Katriona Brooksbank, Linsay McCallum, Christian Delles, Sandosh Padmanabhan, S Faisal Ahmed, Rhian M Touyz

**Affiliations:** Institute of Cardiovascular and Medical Sciences, British Heart Foundation Centre for Research Excellence, University of Glasgow, 126 University Avenue, Glasgow G12 8TA, UK; Developmental Endocrinology Research Group, School of Medicine, Dentistry and Nursing, University of Glasgow, Royal Hospital for Children, 1345 Govan Road, Glasgow G45 8TF, UK; Institute of Cardiovascular and Medical Sciences, British Heart Foundation Centre for Research Excellence, University of Glasgow, 126 University Avenue, Glasgow G12 8TA, UK; Institute of Cardiovascular and Medical Sciences, British Heart Foundation Centre for Research Excellence, University of Glasgow, 126 University Avenue, Glasgow G12 8TA, UK; Institute of Cardiovascular and Medical Sciences, British Heart Foundation Centre for Research Excellence, University of Glasgow, 126 University Avenue, Glasgow G12 8TA, UK; Developmental Endocrinology Research Group, School of Medicine, Dentistry and Nursing, University of Glasgow, Royal Hospital for Children, 1345 Govan Road, Glasgow G45 8TF, UK; Department of Pediatric Surgery, Royal Hospital for Children, 1345 Govan Road, Glasgow G45 8TF, UK; Department of Pediatric Surgery, Royal Hospital for Children, 1345 Govan Road, Glasgow G45 8TF, UK; Department of Pediatric Surgery, Royal Hospital for Children, 1345 Govan Road, Glasgow G45 8TF, UK; Department of Pediatric Surgery, Royal Hospital for Children, 1345 Govan Road, Glasgow G45 8TF, UK; Department of Pediatric Surgery, Royal Hospital for Children, 1345 Govan Road, Glasgow G45 8TF, UK; Institute of Cardiovascular and Medical Sciences, British Heart Foundation Centre for Research Excellence, University of Glasgow, 126 University Avenue, Glasgow G12 8TA, UK; Institute of Cardiovascular and Medical Sciences, British Heart Foundation Centre for Research Excellence, University of Glasgow, 126 University Avenue, Glasgow G12 8TA, UK; Institute of Cardiovascular and Medical Sciences, British Heart Foundation Centre for Research Excellence, University of Glasgow, 126 University Avenue, Glasgow G12 8TA, UK; Institute of Cardiovascular and Medical Sciences, British Heart Foundation Centre for Research Excellence, University of Glasgow, 126 University Avenue, Glasgow G12 8TA, UK; Developmental Endocrinology Research Group, School of Medicine, Dentistry and Nursing, University of Glasgow, Royal Hospital for Children, 1345 Govan Road, Glasgow G45 8TF, UK; Institute of Cardiovascular and Medical Sciences, British Heart Foundation Centre for Research Excellence, University of Glasgow, 126 University Avenue, Glasgow G12 8TA, UK

**Keywords:** Hypospadias, Disorder of sex development, Vessel, Reactive oxygen species, Testosterone

## Abstract

**Aims:**

Hypogonadism is associated with cardiovascular disease. However, the cardiovascular impact of hypogonadism during development is unknown. Using hypospadias as a surrogate of hypogonadism, we investigated whether hypospadias is associated with vascular dysfunction and is a risk factor for cardiovascular disease.

**Methods and results:**

Our human study spanned molecular mechanistic to epidemiological investigations. Clinical vascular phenotyping was performed in adolescents with hypospadias and controls. Small subcutaneous arteries from penile skin from boys undergoing hypospadias repair and controls were isolated and functional studies were assessed by myography. Vascular smooth muscle cells were used to assess: Rho kinase, reactive oxygen species (ROS), nitric oxide synthase/nitric oxide, and DNA damage. Systemic oxidative stress was assessed in plasma and urine. Hospital episode data compared men with a history of hypospadias vs. controls. In adolescents with hypospadias, systolic blood pressure (*P* = 0.005), pulse pressure (*P* = 0.03), and carotid intima-media thickness standard deviation scores (*P* = 0.01) were increased. Arteries from boys with hypospadias demonstrated increased U46619-induced vasoconstriction (*P* = 0.009) and reduced acetylcholine-induced endothelium-dependent (*P* < 0.0001) and sodium nitroprusside-induced endothelium-independent vasorelaxation (*P* < 0.0001). Men born with hypospadias were at increased risk of arrhythmia [odds ratio (OR) 2.8, 95% confidence interval (CI) 1.4–5.6, *P* = 0.003]; hypertension (OR 4.2, 95% CI 1.5–11.9, *P* = 0.04); and heart failure (OR 1.9, 95% CI 1.7–114.3, *P* = 0.02).

**Conclusion:**

Hypospadias is associated with vascular dysfunction and predisposes to hypertension and cardiovascular disease in adulthood. Underlying mechanisms involve perturbed Rho kinase- and Nox5/ROS-dependent signalling. Our novel findings delineate molecular mechanisms of vascular injury in hypogonadism, and identify hypospadias as a cardiovascular risk factor in males.

## Structured Graphical Abstract

**Structured Graphical Abstract:**
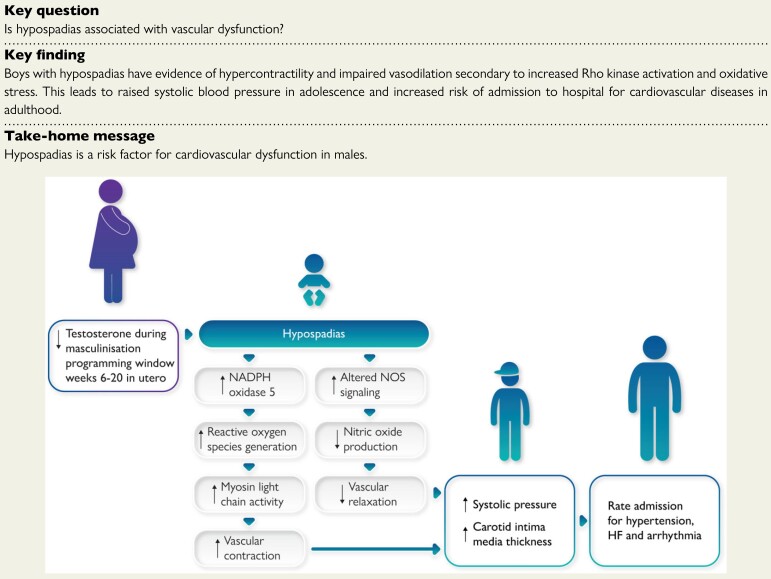
Vascular dysfunction is present in boys with hypospadias. HF, heart failure; NADPH, nicotinamide adenine dinucleotide phosphate; NOS, nitric oxide synthase.

**See the editorial comment for this article ‘Increased cardiovascular risk in boys born with hypospadias: intriguing observations and remaining questions’, by Nicolle Kränkel, https://doi.org/10.1093/eurheartj/ehac152.**

## Introduction

Androgens have crucial extragonadal cardiovascular effects, including regulation of vascular contraction and relaxation.^1^ Hypogonadism is defined biochemically as a low circulating testosterone concentration^2^ and has been identified as an independent determinant of endothelial dysfunction in men.^3^ Clinical studies have demonstrated links between hypogonadism in adult men and morbidity and mortality secondary to cardiovascular disease (CVD), the leading cause of death worldwide.^4,5^ Molecular and cellular mechanisms whereby testosterone deficiency/hypogonadism affects vascular function are complex and involve multiple androgen-sensitive signalling pathways. In particular, testosterone influences vascular reactivity by regulating vascular smooth muscle cell (VSMC) Ca^2+^ channel expression and activity, intracellular Ca^2+^ homeostasis, Nox-derived reactive oxygen species (ROS) generation, nitric oxide (NO) production, Rho kinase activation, and mitogen-activated protein kinase phosphorylation.^6–9^

Pre-clinical studies demonstrated that there is a masculinization programming window during development when androgens are required for normal masculinization of the genital tract.^10^ Lack of androgens during this critical window affects development of the normal penis and may manifest as hypospadias, defined as the abnormal positioning of the urethral meatus in boys.^11^ Most 46,XY boys with genital abnormalities such as hypospadias have normal testosterone levels in childhood;^12^ however boys with hypospadias tend to have shorter anogenital distance (AGD) and anoscrotal distance, which are indicators of reduced antenatal androgen exposure.^13^ Given that boys with hypospadias lack androgens during a critical period of foetal development, during which time the vasculature is also developing, we hypothesized that boys born with hypospadias would have evidence of early onset vascular dysfunction.

Our study spanned the use of molecular mechanisms of vascular dysfunction to big data in a patient cohort. More specifically, we assessed first whether vascular function and structure are altered in adolescents with a history of hypospadias. We then studied isolated small arteries and primary culture VSMCs from young children with hypospadias. We focused on molecular and cellular mechanisms whereby testosterone impacts vascular function because hypospadias is associated with reduced androgen exposure in utero. Finally, we sought to determine whether adult men with a history of hypospadias have an increased risk of CVD using data-linkage analysis.

## Methods

Detailed methods are described in Supplementary material online.

### Ethics approval

Ethics approval was obtained for all studies, as detailed in Supplementary material online.

### Non-invasive vascular phenotyping in adolescents with hypospadias

Boys aged 12.0–18.9 years were recruited from endocrine or urology clinics at the Royal Hospital for Children, Glasgow. Cases were defined as boys born with hypospadias and controls were defined as those with no history of hypospadias. Boys were excluded from recruitment if they had any other health problem or were prescribed any medication, which may alter their vascular status, as detailed in Supplementary material online, *Methods*. In total, 28 boys were included [median age 13 (range 12, 19) years]. Of these, 14 (50%) had a history of proximal hypospadias and 14 (50%) were controls. The clinical characteristics and hormonal profiles of the two groups are shown in Supplementary material online, *Table S1*. All cases of hypospadias had undergone genetic testing using our local 56 gene targeted Disorders of Sex Development gene panel (Supplementary material online, *Methods*), with no variants detected.

#### Blood pressure

Blood pressure was measured by standard sphygmomanometer methods and standard deviation scores (SDSs) were calculated according to gender, height, and age.^14^

#### Carotid intima-media thickness

Carotid intima-media thickness (CIMT), a measure of vascular structure, was assessed in the common carotid arteries using the Acuson Sequoia C512 ultrasound. Carotid intima-media thickness SDSs were calculated based on published reference data.^15^

#### Flow-mediated dilatation

Flow-mediated dilatation (FMD), a marker of endothelial function and vasoreactivity, of the brachial artery was measured in fasted subjects using the UNEX EF machine.

#### Pulse wave analysis and pulse wave velocity

Pulse wave analysis (PWA) and carotid femoral pulse wave velocity (PWV), measures of vascular stiffness and distensibility, were measured using the SphygmoCor XCEL device. Pulse wave velocity SDSs were calculated according to age and height using reference data.^16^

#### Questionnaires

To adjust for physical fitness and health-related quality of life which may affect vascular status, all participants were asked to complete the KIDSCREEN-52 questionnaire for self-perceived quality of life,^17^ and the Physical Activity Questionnaire for Adolescents^18^ was used to assess physical activity.

### Vascular reactivity and molecular and cellular studies in young boys with hypospadias

To identify if there was any evidence of vascular dysfunction in young boys with hypospadias, excess foreskin tissue was obtained from boys undergoing routine urological surgery. Cases were defined as boys undergoing hypospadias repair and controls were defined as boys undergoing circumcision. Samples were only included if they were obtained from surgery using caudal analgesia and samples were taken from the same area of the foreskin in both groups. Boys were excluded from recruitment if they had any associated abnormality or were prescribed any medication, which may alter their vascular status (see Supplementary material online, *Methods*). Subcutaneous arteries were dissected from the skin and VSMCs cultured as we previously described.^19^ Twenty-seven boys with hypospadias (cases) and 37 controls with a median age of 2 years (range 0.8, 12.9) entered the study. The clinical characteristics and fasting blood profile results of the two groups are shown in Supplementary material online, *Table S2*. All cases of proximal hypospadias had undergone genetic testing using our local targeted Disorders of Sex Development gene panel (Supplementary material online, *Methods*), with no variants detected.

#### Wire myography to assess vascular function

Small subcutaneous arteries were mounted on wire myographs (AD Instruments, UK) for vascular reactivity studies (contraction, endothelium-dependent vasorelaxation, and endothelium-independent vasorelaxation) as we previously described.^20^ Optimization of this technique in paediatric vessels including studies into any differences between ethnicity and birthweight is discussed in Supplementary material online, *Methods* and specifically Supplementary material online, *Figures S1* and *S2*. Constriction curves were conducted using cumulative increasing doses of the thromboxane A2 analogue U46619 (1 × 10^−10^–3 × 10^−6^ M), as this was the most consistent vasoconstrictor in these arteries, as demonstrated in Supplementary material online, *Figure S2B*. Endothelium-dependent relaxation was assessed using relaxation curves to acetylcholine (Ach) (1 × 10^−9^−3 × 10^−5^ M) and endothelium-independent vasodilation was assessed using sodium nitroprusside (SNP) (1 × 10^−9^−3 × 10^−5^ M).

### Vascular smooth muscle cell protocols

Low passage VSMCs (P3–6) from controls and hypospadias subjects were studied and the following parameters examined, in line with possible actions of androgens on the vasculature: mRNA expression, calcium signalling, ROS generation, Rho kinase activity, protein expression, NO and peroxynitrite levels, DNA methyltransferase (DNMT) activity, and thiobarbituric acid reactive substances (TBARS). Details are provided in Supplementary material online, *Methods*.

#### Quantitative real-time polymerase chain reaction

mRNA expression of *Rho GEF P115*, *Rho GEF PDZ*, *LARG*, *iNOS*, *eNOS*, *nNOS*, *SOD1*, *TBXA2R*, *CACNA1C*, *IP3R*, *TRPM2*, *SERCA*, *RyR1*, *RyR2*, and *RyR3* was measured in VSMCs using quantitative real-time polymerase chain reaction.

#### Immunoblotting

Vascular smooth muscle cell proteins were extracted and separated by electrophoresis and transferred onto a nitrocellulose membrane. Membranes were incubated with antibodies and fluorescent signals measured.

#### Calcium signalling

Fluorescent measurement of the Ca^2+^ indicator Cal-520 acetoxymethyl ester (Cal-520/AM, Abcam, 10 µmol/L) was used to identify differences in Ca^2+^ transients in VSMCs from boys with hypospadias and controls.

#### Oxidative stress

Reactive oxygen species generation in VSMCs was assessed using lucigenin-enhanced chemiluminescence, Amplex Red, and electron paramagnetic resonance (EPR) techniques. Total antioxidant capacity was measured in urine.

#### Rho kinase activity

Rho kinase levels were assessed in cell lysates using a Rho kinase activity kit as per the manufacturer’s instructions.

#### Nitric oxide and peroxynitrite

Nitric oxide was measured in cell lysates with a 0.3 mM DAF-FM diacetate (4-amino-5-methylamino-2′,7′-difluorofluorescein diacetate) probe. Peroxynitrite levels in VSMC lysates were measured using a commercial kit.

#### Markers of systemic oxidative stress: thiobarbituric acid reactive substances and urinary levels of 8-hydroxy 2-deoxyguanosine

Thiobarbituric acid reactive substances were measured in plasma to assess lipid peroxidation and 8-hydroxy 2-deoxyguanosine (8OH-dG), a marker of oxidative DNA damage, were assessed by commercial ELISA kits.

#### DNA methyltransferase activity

DNA methyltransferase activity was measured in VSMCs as a marker of epigenetic changes by ELISA.

### Data-linkage studies: relationship between hypospadias and cardiovascular disease in adult men

We used a large Scottish data set to link hypospadias with CVD. In Scotland, data on all National Health Service (NHS) encounters have been routinely collected since January 1981, using the Information Services Division (ISD) Scottish Morbidity Record (SMR) Scheme. At the time of presentation or medication prescription, hospital software is used to code the underlying diagnosis using the World Health Organisation International Classification of Diseases (ICD-9 before 1996 and ICD-10 after 1996) system. Pseudo-anonymized data were then obtained from ISD for a case–control cohort. The cases were all men above the age of 18 years at the time of data collection with an ICD-10 code of Q54 for hypospadias and controls were matched to sex, age, gestation, birthweight, and the Scottish Index of Multiple Deprivation (SIMD), as detailed in Supplementary material online, *Methods*. Supplementary material online, *Figure S7* demonstrates the numbers of records identified. The SIMD is an index of deprivation that takes into account data on employment, average income, health, education, housing, crime, and access to local services. The lower the number, the more socially deprived the geographical region.^21^ Men were excluded if they had a history of congenital heart disease. Details of the two groups are shown in Supplementary material online, *Table S5* and all data on outcomes are displayed in Supplementary material online, *Table S6*.

### Statistical analysis

Statistical analysis was performed using the GraphPad PRISM 8 software (GraphPad Software Inc., San Diego, CA, USA), R version R.3.6.3 (R Foundation for Statistical Computing, USA), and SPSS version 22.0 (IBM, Armonk, NY, USA). Details can be found in Supplementary material online. A value of *P* < 0.05 was deemed statistically significant.

## Results

### Increased systolic blood pressure and carotid intima-media thickness in adolescents with hypospadias

**Figure 1:**
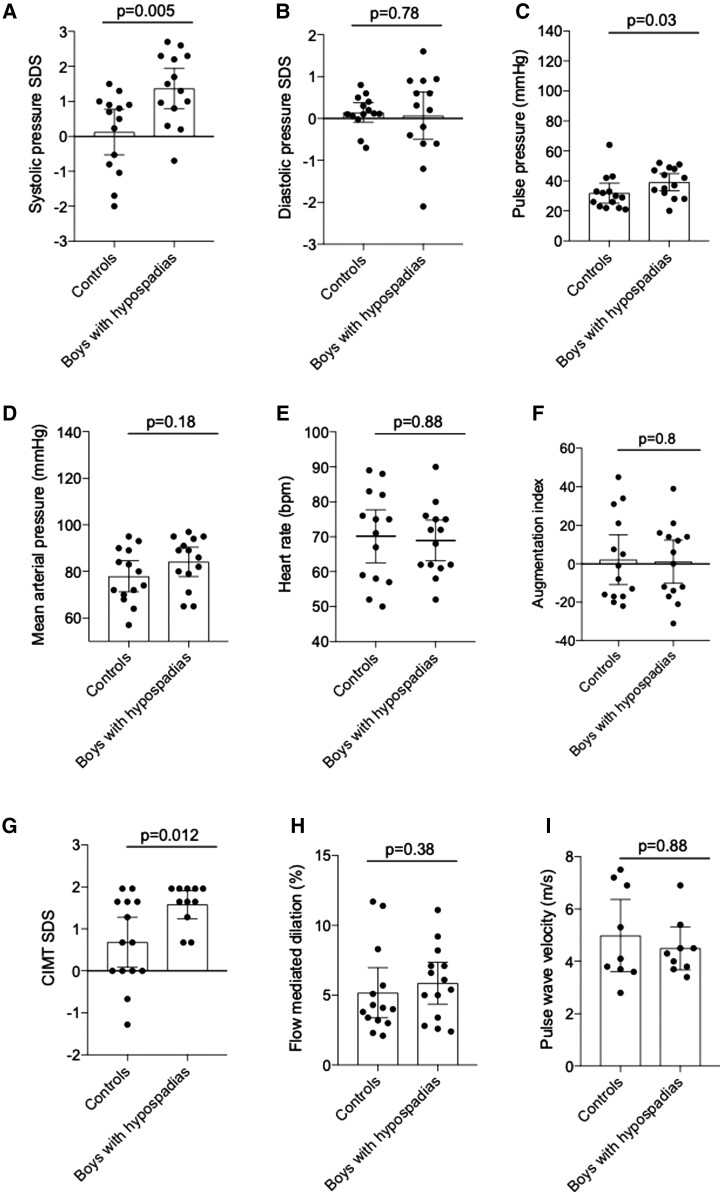
Clinical vascular phenotyping in boys with hypospadias and controls. Systolic blood pressure standard deviation score (*A*) and pulse pressure (*C*) were increased in boys with hypospadias compared with controls, but there were no differences in diastolic blood pressure standard deviation score (*B*), mean arterial pressure (*D*), heart rate (*E*), or augmentation index (*F*). Carotid intima-media thickness standard deviation score (*G*) was also increased in boys with hypospadias, but there were no differences in flow-mediated dilatation (*H*) or pulse wave analysis (*I*). Results are mean ± 95% CI of 14 cases and controls. Data were analysed by Mann–Whitney *U* test. BP, blood pressure; CIMT, carotid intima-media thickness; FMD, flow-mediated dilatation; MAP, mean arterial pressure; PWA, pulse wave analysis; PWV, pulse wave velocity; SDS, standard deviation score.

Systolic blood pressure SDS was significantly higher in boys with hypospadias with a median (range) of 1.4 (−0.7, 2.7) compared with 0.2 (−0.7, 0.8) in controls (*P* = 0.005) (*Figure 1A*). There was no statistically significant difference in diastolic blood pressure between boys with hypospadias and controls (*Figure 1B*), but pulse pressure was significantly higher in cases with a median (range) of 40 mmHg (20, 52) compared with 26 mmHg (21, 64) in controls (*P* = 0.03) (*Figure 1C*). There were no significant differences between groups in mean arterial pressure (*Figure 1D*), heart rate (*Figure 1E*), or augmentation index as measured by pulse wave analysis (*Figure 1F*).

Vascular structural changes were assessed by measuring CIMT in the groups as a whole and according to age. As shown in *Figure 1G*, CIMT SDS calculated according to the age of the participant was greater in boys with hypospadias compared with controls [median 1.6 (range 0.7, 2.0) vs. 1.2 (range −1.2, 2.0) (*P* = 0.012)]. There was no difference in absolute CIMT values between the two groups, with a median (range) of 0.05 cm (0.04, 0.06) in cases and 0.04 cm (0.04, 0.06) in controls (*P* = 0.9).

Vascular function was evaluated by measuring FMD, with no differences between the two groups, with a median (range) of 5.7% (2.4, 11.1) in boys with hypospadias and 4.1% (2.1, 11.7) in controls (*P* = 0.38) (*Figure 1H*). Baseline vascular diameter [median 3.2 mm in boys with hypospadias (range 1.4, 4.6) vs. 3.0 mm in controls (range 1.3, 4.2) (*P* = 0.4)] and maximal diameter [median 3.9 in boys with hypospadias (range 2.5, 5.0) vs. 3.2 in controls (range 1.6, 4.5) (*P* = 0.09)] were not significantly different between groups.

Pulse wave velocity, a measure of vascular stiffness, was not significantly different between the two groups, with a median (range) of 4.3 (3.4, 6.9) m/s in boys with hypospadias and 3.9 (2.8, 7.5) m/s in controls (*P* = 0.88) (*Figure 1I*).

### Vascular dysfunction in boys with hypospadias

As shown in Supplementary material online, *Figure S3A*, U46619-induced vasoconstriction was significantly increased in cases compared with controls (*E*_max_ % KCl: 137.9 vs. 83.7 *P* = 0.009). There were no significant differences in mRNA levels of *TBXA2R* in VSMCs from boys with hypospadias and controls (see Supplementary material online, *Figure S4*). Endothelium-dependent relaxation in response to ACh and endothelium-independent relaxation in response to SNP were reduced in arteries from cases vs. controls (*E*_max_ % KCl: 77.3 vs. 14.6, *P* < 0.0001 and *E*_max_ % KCl: 39.5 vs. 24.6, *P* < 0.0001, respectively) (see Supplementary material online, *Figures S3B* and *C*).

### Role of Rho kinase and redox-dependent processes

**Figure 2:**
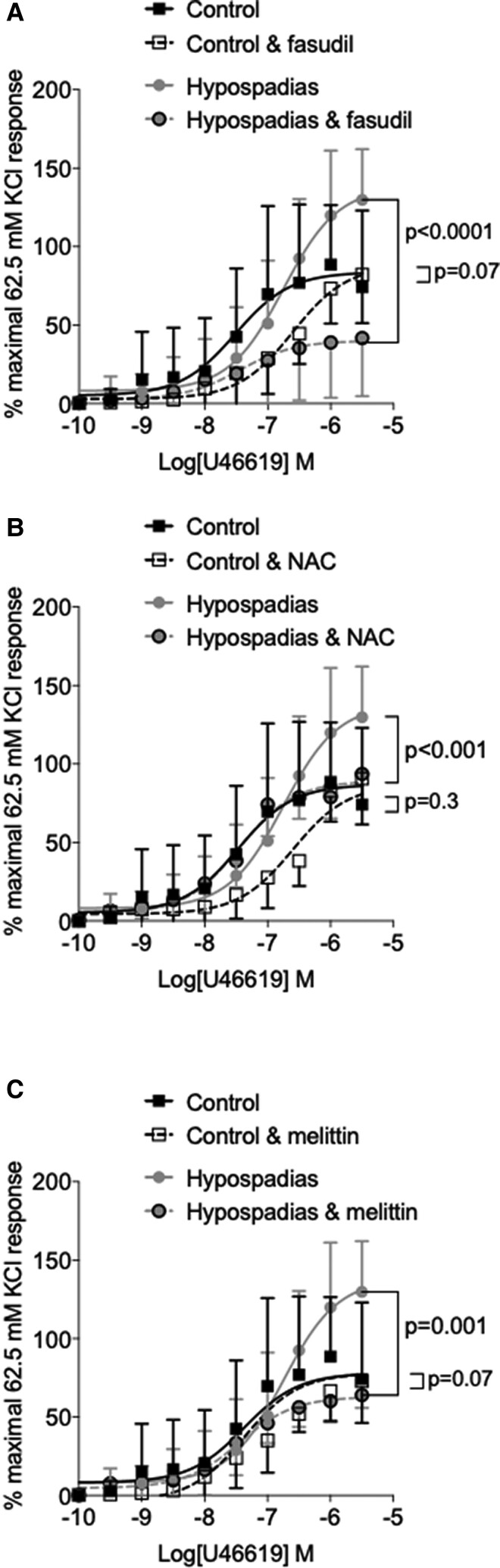
Mechanisms of hypercontractility in boys with hypospadias. (*A*) Fasudil significantly reduced contraction in arteries from cases, but not from controls. (*B*) *N*-acetylcysteine significantly reduced contraction in arteries from cases but had no effect on controls. (*C*) The Nox5 inhibitor, melittin, significantly reduces contraction in arteries from boys with hypospadias, but not controls. Results are mean ± 95% confidence interval of blood vessels from six cases and nine controls. When >1 blood vessel was obtained from the same boy, the mean was used. Best fit cumulative concentration curves were compared with the extra sum-of-squares *F*-test. Student’s *t*-test was used to calculate differences in maximum response. KCl, potassium chloride; NAC, *N*-acetylcysteine.

To evaluate potential mechanisms underlying hypercontractile responses and impaired vasorelaxation, we examined the role of Rho kinase, ROS, and eNOS/NO, which have been shown to be important in testosterone-mediated vascular regulation in experimental models.^5,8,22,23^ In addition, we probed for Nox5, which we previously showed was an important pro-contractile Nox isoform.^24^ Vessels were pre-treated with fasudil (Rho kinase inhibitor), *N*-acetylcysteine (NAC) (ROS scavenger), l-NAME (eNOS inhibitor), or melittin (Nox5 inhibitor). As demonstrated in *Figure 2A*, hypercontractile responses in cases were attenuated in vessels pre-treated with the Rho kinase inhibitor fasudil compared with vessels treated with vehicle (*E*_max_ % KCl: 40.1 vs. 137.9, *P* < 0.0001, fasudil vs. vehicle). Fasudil had no significant effect on maximal contraction in vessels from control subjects (*E*_max_ % KCl: 72.8 vs. 83.7, *P* = 0.07; fasudil vs. vehicle). In the presence of NAC, contractile responses to U46619 were unchanged in controls (*E*_max_ % KCl: 90.7 vs. 83.7, *P* = 0.3) but significantly reduced in vessels from boys with hypospadias (*E*_max_ % KCl: 137.9 vs. 89.1, *P* = 0.01) (*Figure 2B*). When arteries were incubated with melittin, contraction in arteries from cases (*E*_max_ % KCl: 137.9 vs. 62.9, *P* < 0.0001) was normalized (*Figure 2C*). Melittin did not significantly influence vascular contractile responses in arteries from control subjects (*E*_max_ % KCl: 83.7 vs. 72.7, *P* = 0.07).

**Figure 3:**
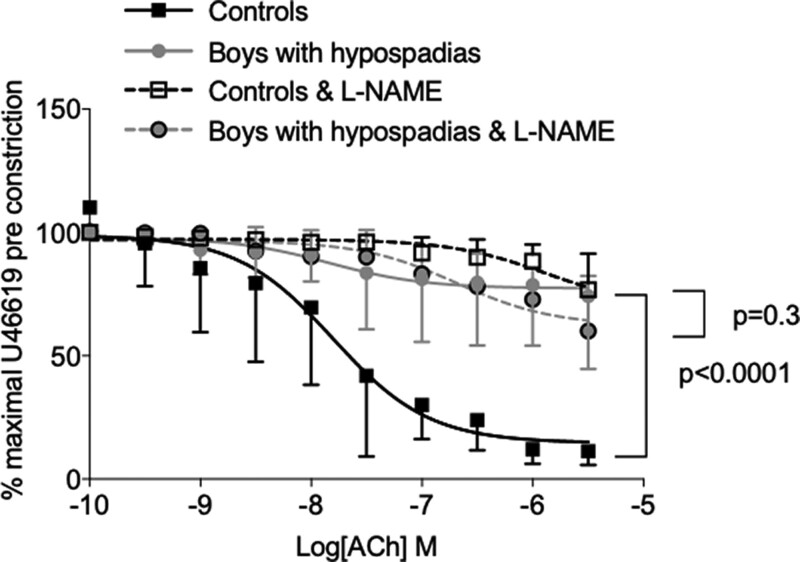
Effects of nitric oxide inhibition in arteries from boys with hypospadias. (*A*) Nitric oxide inhibition via l-NAME did not alter endothelial-dependent vasodilation in arteries from boys with hypospadias but increased endothelial-dependent vasodilatation in arteries from controls. Results are mean ± 95% confidence interval of blood vessels from six cases and nine controls. When >1 blood vessel was obtained from the same boy, the mean was used. Best fit cumulative concentration curves were compared with the extra sum-of-squares *F*-test. Student’s *t*-test was used to calculate differences in maximum response. Ach, acetylcholine; NO, nitric oxide; SNP, sodium nitroprusside.

To investigate the role of eNOS/NO in vasodilation, vessels were exposed to l-NAME. In vessels pre-treated with l-NAME, endothelium-dependent vasorelaxation in controls was reduced (*E*_max_ % U46619: 76.8 vs. 1.2, *P* < 0.0001) without effect in cases (*E*_max_ % U46619: 60.6 vs. 72.4, *P* = 0.3) (*Figure 3*).

### Impaired vascular smooth muscle cell signalling in hypospadias

**Figure 4:**
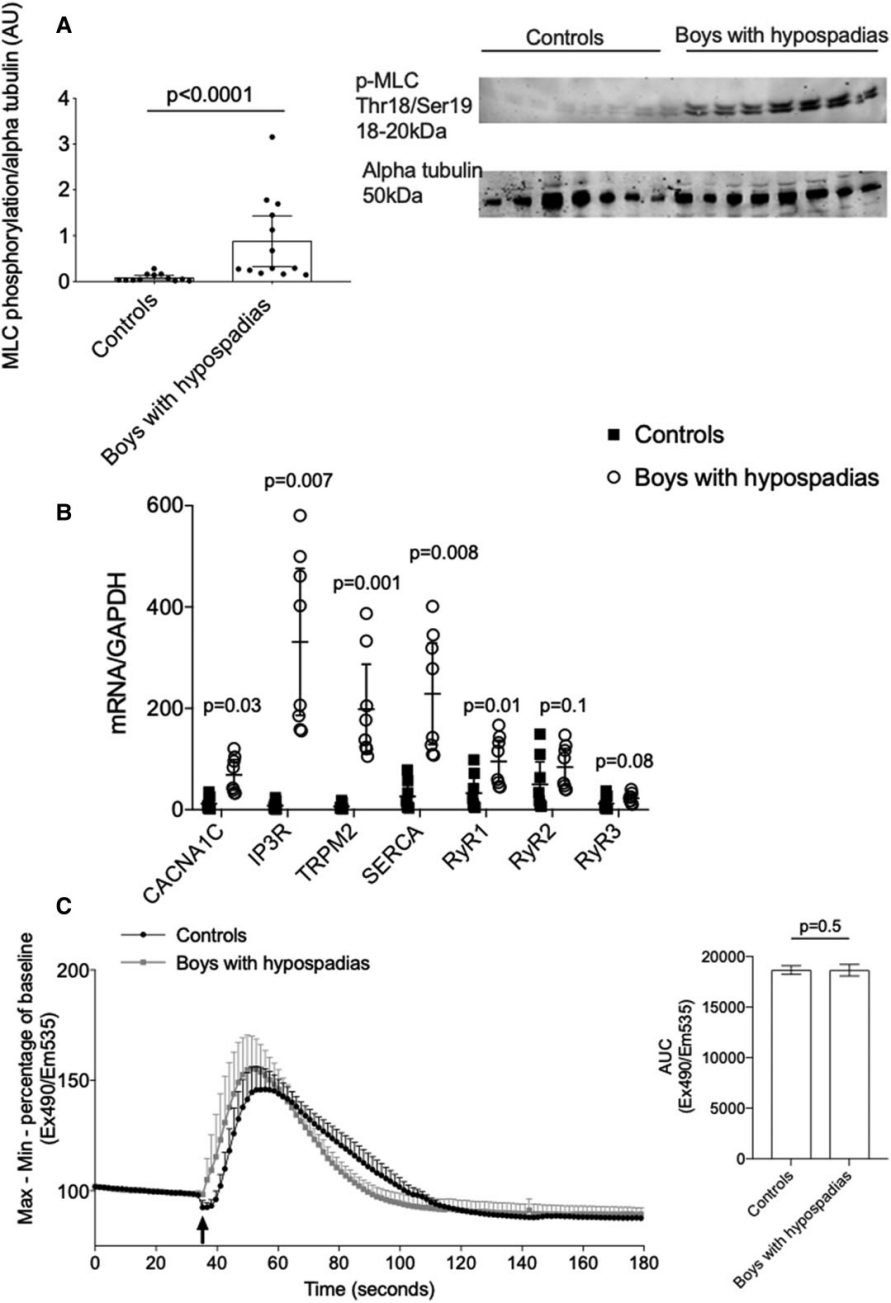
Mechanisms of contraction in vascular smooth muscle cells from boys with hypospadias. (*A*) Vascular smooth muscle cells from boys with hypospadias had significantly increased myosin light chain phosphorylation compared with controls. Results are mean ± 95% confidence interval of 13 controls and 13 boys with hypospadias. Data were analysed by Mann–Whitney *U* test. (*B*) Vascular smooth muscle cells from boys with hypospadias had increased expression of *CACNA1C*, *IP3R*, *TRPM2*, *SERCA*, and *RyR1* compared with controls. Results are from 10 controls and 10 boys with hypospadias. Data were analysed using the Wilcoxon signed-rank test. (*C*) At baseline, there was no difference in Ca^2+^ influx between VSMCs of boys with hypospadias and controls in response to 10^−6^ M U46619 (arrow) as shown by the Ca^2+^ signalling trace and area under the curve. Results are mean ± 95% confidence interval of five controls and five boys with hypospadias. AUC, area under the curve; MLC, myosin light chain.

To investigate some molecular mechanisms underlying increased vasoreactivity in hypospadias, we examined key signalling elements involved in VSMC contraction. Phosphorylation of the pro-contractile signalling molecule MLC was significantly increased in VSMCs from cases vs. controls (*Figure 4A*). This was associated with increased mRNA expression of Ca^2+^ channels important in regulating VSMC Ca^2+^ influx, specifically *CACNA1C* (*P* = 0.03), *IP3R* (*P* = 0.007), *TRPM2* (*P* = 0.001), *SERCA* (*P* = 0.008), and *RyR1* (*P* = 0.01) in boys with hypospadias vs. controls (*Figure 4B*). An important trigger in vasoconstriction is an increase in VSMC Ca^2+^ levels. As shown in *Figure 4C*, U46619-induced robust Ca^2+^ transients in VSMCs from cases and controls, without significant differences between groups.

**Figure 5:**
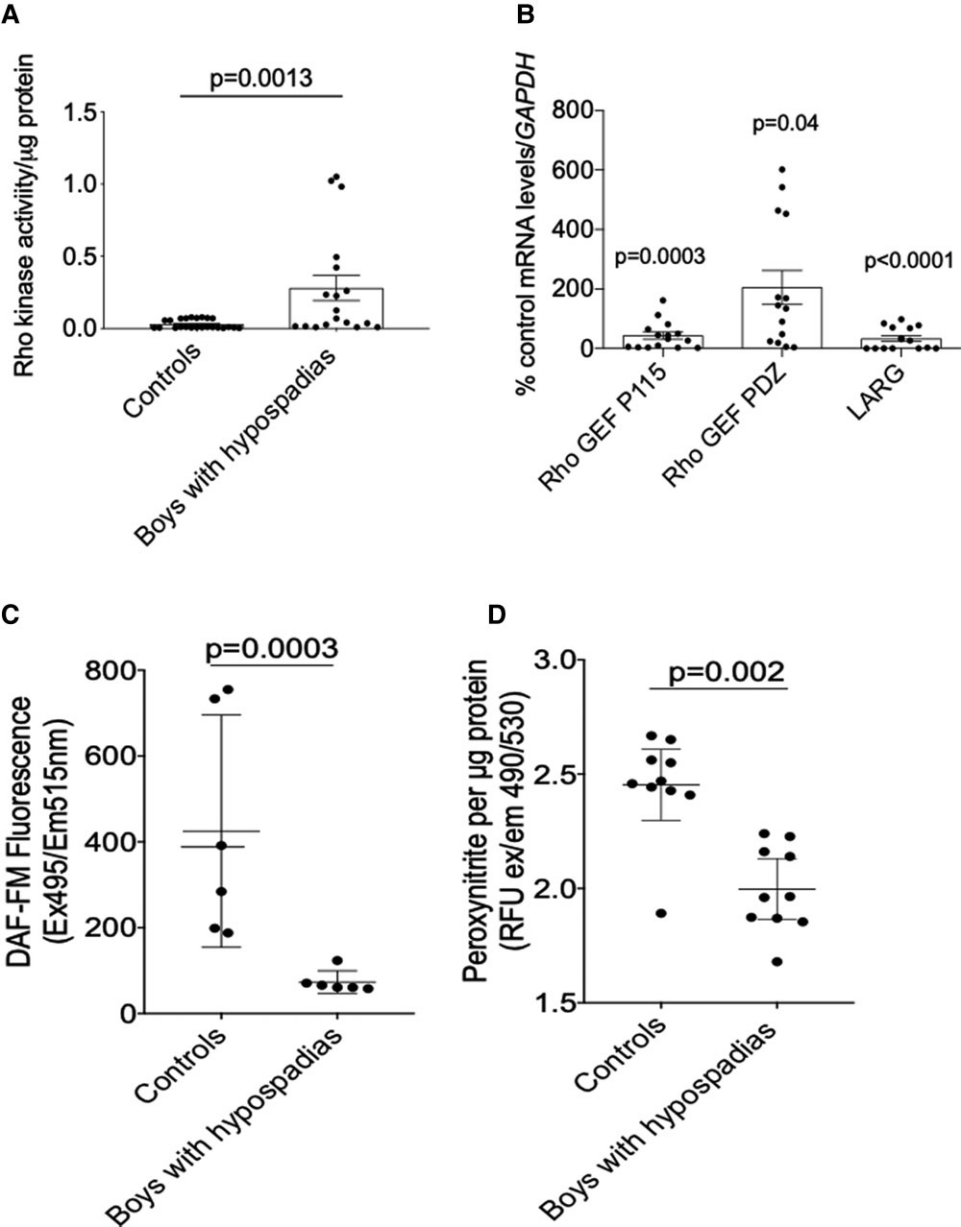
Rho kinase and nitric oxide signalling in vascular smooth muscle cells from boys with hypospadias. (*A*) Vascular smooth muscle cells from cases had increased Rho kinase activity compared with controls. Results are mean ± 95% confidence interval of 21 controls and 19 cases. Data were analysed by Mann–Whitney *U* test. (*B*) mRNA receptor expression of the Rho GEF genes in cases vs. controls. Cases had increased expression of Rho GEF PDZ and reduced expression of Rho GEF P115 and LARG. Results are shown as percentage of control from 18 controls and 15 cases. Data were analysed using the Wilcoxon signed-rank test. (*C* and *D*) Boys with hypospadias had reduced nitric oxide (*C*) and reduced peroxynitrite generation (*D*) compared with controls. Results are percentage of control or mean ± 95% confidence interval of vascular smooth muscle cells from 6 to 10 cases and controls. Data were analysed by the Mann–Whitney *U* test. NO, nitric oxide; VSMC, vascular smooth muscle cell.

Mechanisms that regulate VSMC contraction in a Ca^2+^-independent manner involve RhoA/Rho kinase, which sensitizes the contractile machinery to intracellular Ca^2+^. As shown in *Figure 5A*, Rho kinase activity was significantly increased in VSMCs from hypospadias patients compared with controls (*P* = 0.0013). In addition, boys with hypospadias had statistically significantly increased mRNA expression of the regulatory Rho molecule *Rho GEF PDZ* (*P* = 0.04) but not of *Rho GEF p115* (*P* = 0.0003) or *Rho GEF LARG* (*P* < 0.0001) (*Figure 5B*).

The nitric oxide synthase (NOS)/NO pathway is critically involved in the regulation of vasorelaxation. Vascular smooth muscle cells express NOS isoforms *eNOS*, *iNOS*, and *nNOS* as shown in Supplementary material online, *Figure S5*. Boys with hypospadias had reduced expression of *iNOS* (34.5-fold, *P* = 0.0039) (see Supplementary material online, *Figure S5A*) and *eNOS* (58.8-fold, *P* = 0.007) (see Supplementary material online, *Figure S5B*) but increased expression of *nNOS* (3.0-fold, *P* = 0.008) (see Supplementary material online, *Figure S5C*) compared with controls. Nitric oxide bioavailability was reduced in cases (five-fold, *P* = 0.0003) (*Figure 5C*), as was peroxynitrite production (0.5-fold, *P* = 0.002) (*Figure 5D*).

### Increased vascular oxidative stress in hypospadias

**Figure 6:**
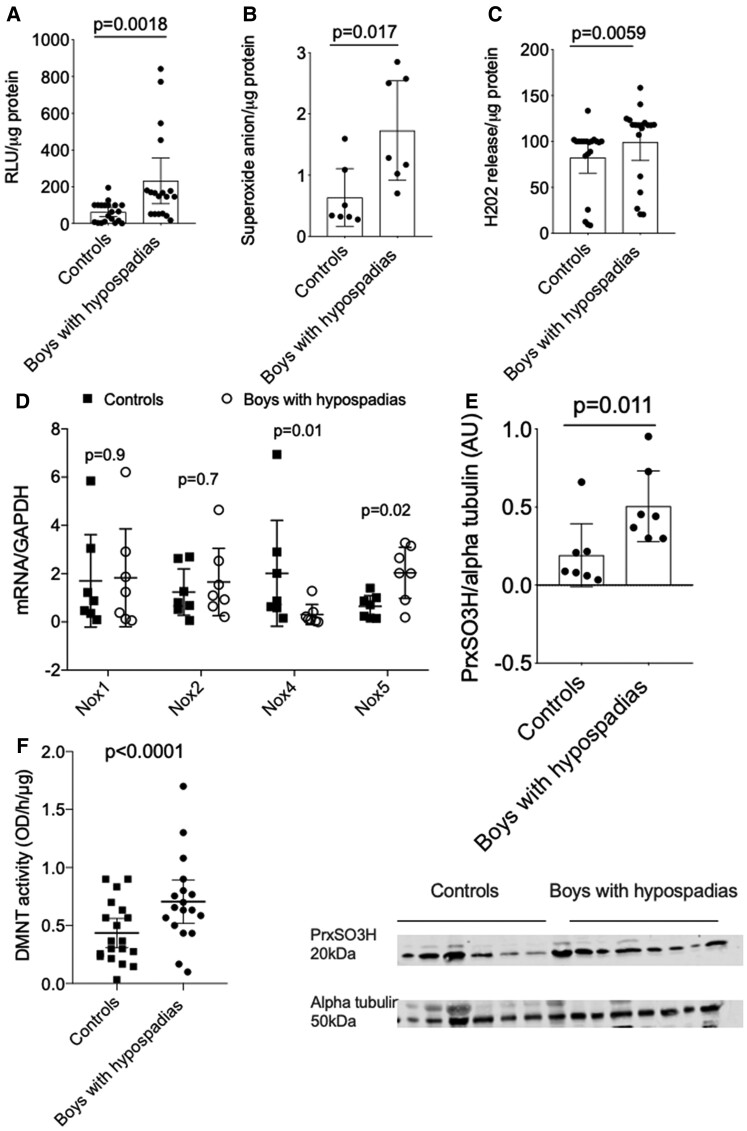
Reactive oxygen species generation and NADPH oxidase expression in vascular smooth muscle cells from boys with hypospadias and controls. (*A–C*) Vascular smooth muscle cells from cases had significantly increased levels of superoxide as measured by lucigenin (*A*), electron paramagnetic resonance (*B*) and H_2_O_2_ generation (*C*) compared with controls. Results are mean ± 95% confidence interval of 21 controls and 19 cases (*A* and *C*) and 7 controls and cases (*B*). Data were analysed using the Mann–Whitney *U* test. (*D*) Vascular smooth muscle cells from boys with hypospadias had reduced mRNA expression of Nox4 and increased mRNA expression of Nox5 compared with controls (*D*). Results are shown from eight controls and eight boys with hypospadias. Data were analysed using the Wilcoxon signed-rank test. (*E*) Boys with hypospadias had increased irreversible protein oxidation compared with controls. Results are mean ± 95% confidence interval of seven controls and seven boys with hypospadias. (*F*) Boys with hypospadias have increased DNA methyltransferase activity compared with controls. Results are mean ± 95% confidence interval of 19 controls and 19 cases. Data were analysed using the Wilcoxon signed-rank test. DNMT, DNA methyltransferase; PrxSO3H, sulphonated peroxiredoxin.

Basal NADPH-derived ROS generation as measured by lucigenin-enhanced chemiluminescence (*P* = 0.0018) (*Figure 6A*) and superoxide levels measured by EPR (*P* = 0.017) (*Figure 6B*) were significantly increased in boys with hypospadias compared with controls. In addition, H_2_O_2_ production assessed by Amplex Red was higher in VSMCs from boys with hypospadias (*P* = 0.0059) (*Figure 6C*). To investigate the role of ROS-generating oxidases, mRNA expression of Nox isoforms was measured. As demonstrated in *Figure 6D*, expression of Nox4 was significantly reduced (*P* = 0.01) and that of Nox5 increased in VSMCs from cases vs. controls (*P* = 0.02). Vascular smooth muscle cell expression of Nox1 and Nox2 was not different between groups (*Figure 6D*). Inhibition of NOS via l-NAME reduced superoxide production in VSMCs from boys with hypospadias (*P* = 0.007) (see Supplementary material online, *Figure S7*).

An important consequence of increased oxidative stress is post-translational oxidative modification of effector signalling molecules. Oxidation may be reversible or irreversible, with irreversible oxidation leading to cell injury and death. We assessed irreversible protein oxidation by measuring oxidation of peroxiredoxin by immunostudies. As shown in *Figure 6E*, VSMCs from boys with hypospadias had increased peroxiredoxin oxidation compared with controls (*P* = 0.02). DNA methyltransferase activity, which is a marker of epigenetic modification and associated with oxidative damage, was measured in VSMCs from boys with hypospadias and controls. DNA methyltransferase activity was increased 1.3-fold (*P* < 0.0001) compared with controls in VSMCs from boys with hypospadias (*Figure 6F*). Associated with increased ROS bioavailability in VSMCs from boys with hypospadias was reduced VSMC expression of antioxidant genes. As shown in Supplementary material online, *Figure S6*, VSMCs from cases had reduced mRNA expression of *SOD1* compared with VSMCs from controls (5.5-fold, *P* < 0.0001).

### Increased markers of systemic oxidative stress in hypospadias

**Figure 7:**
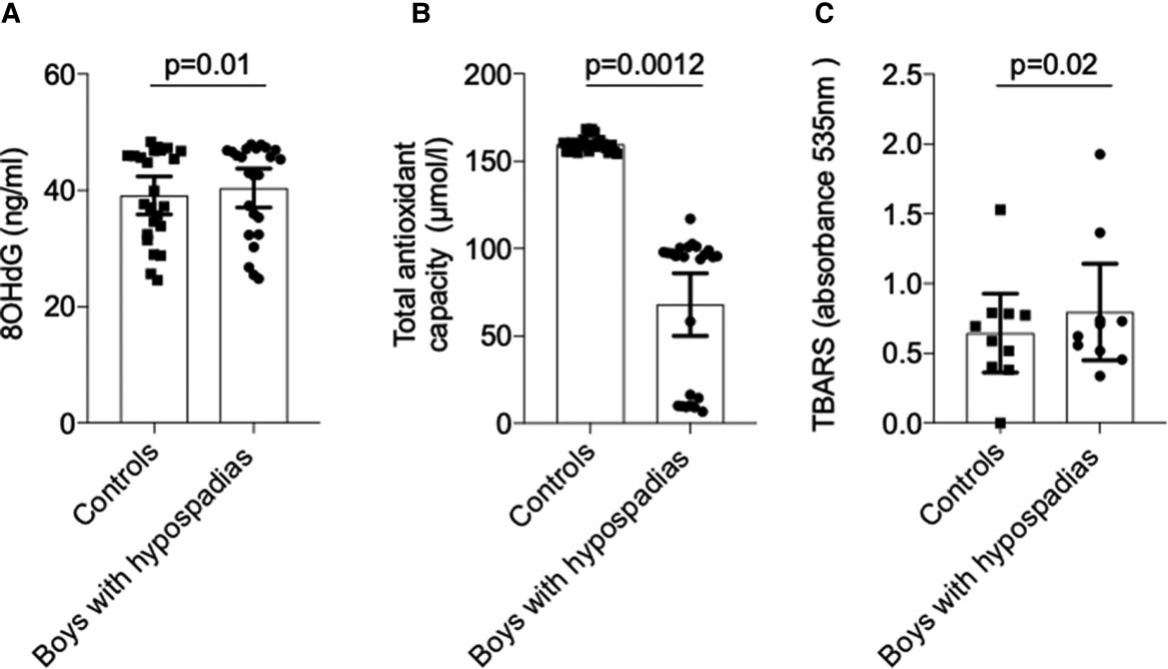
Oxidative stress and DNA methylation in boys with hypospadias and controls. (*A*) Free 8-hydroxy 2-deoxyguanosine levels were increased in urine from boys with hypospadias compared with controls. Results are mean ± 95% confidence interval from 24 controls and 24 cases. Data were analysed by the Mann–Whitney *U* test. (*B*) Cases had reduced total antioxidant capacity compared with controls. Results are mean ± 95% confidence interval from 24 controls and 24 cases. Data were analysed by the Mann–Whitney *U* test. (*C*) Thiobarbituric acid reactive substances concentration was increased in the plasma of cases compared with controls. Results are mean ± 95% confidence interval of 10 controls and 10 boys with hypospadias. Data for all were analysed by the Wilcoxon signed-rank test. 8OH-dG, 8-hydroxy 2-deoxyguanosine; TAOC, total antioxidant capacity; TBARS, thiobarbituric acid reactive substances.

As shown in *Figure 7A*, urinary levels of 8-OHdG, an oxidatively modified guanosine used as a marker of oxidative DNA damage,^25^ were increased in boys with hypospadias vs. controls (*P* = 0.01). Plasma total antioxidant capacity was reduced in cases with hypospadias (*P* = 0.0012) (*Figure 7B*).

Systemic lipid peroxidation, assessed by measuring plasma TBARS levels, was increased in boys with hypospadias (*P* < 0.0001) (*Figure 7C*).

### Men born with hypospadias have an increased risk of cardiovascular disease: arrhythmias, heart failure, and hypertension

To assess whether the vascular dysfunction seen in our studies above in childhood and adolescence affects adult cardiovascular risk, a cohort of 8073 controls and 6797 cases were analysed (see Supplementary material online, *Figure S8*). The median (range) age of the men was 29 years (18, 38) for controls and 23 (18, 38) years for cases (*P* = 0.2). There were no significant differences in birthweight, gestation, SIMD, or frequency of maternal diabetes between groups. 165/8073 (2.3%) controls and 462/6797 (6.7%) cases (*P* < 0.0001) had a total of 189 and 1728 admissions for cardiovascular diagnoses, respectively [median number per person (range) 0 (0, 173) and 1 (0, 54), *P* = 0.07].

Men born with hypospadias were at increased risk of arrhythmia [odds ratio (OR) 2.8, 95% confidence interval (CI) 1.4–5.6, *P* = 0.003]; hypertension (OR 4.2, 95% CI 1.5–11.9, *P* = 0.04), and heart failure (OR 1.9, 95% CI 1.7–114.3, *P* = 0.02) on multivariable analysis adjusting for birthweight, gestation, SIMD, maternal smoking during pregnancy, maternal diabetes during pregnancy and antenatal steroids (*Table 1*). Data on frequency of all cardiovascular outcomes are shown in Supplementary material online, *Table S6*. Based on the data and coding, we could not discern the type of arrhythmia or whether the cases with heart failure represented heart failure with preserved or reduced ejection fraction.

**Table 1:** Univariable and multivariable regression of admission for cardiometabolic diseases in 6797 men with hypospadias compared with 8073 controls

Admission diagnosis	No. of admissions of men with hypospadias (%)	No. of admissions of controls (%)	Univariable OR	95% CI	*P*-value	Multivariable OR	95% CI	*P*-value
**Arrhythmia**	36 (0.5)	16 (0.2)	2.5	1.4–4.6	0.003	2.8	1.4–5.6	0.003*
**Diabetes**	71 (0.9)	65 (0.8)	1.5	0.9–2.5	0.09	1.5	0.8–2.6	0.15
**Hypertension**	51 (0.7)	14 (0.2)	2.3	0.9–5.8	0.05	4.2	1.5–11.9	0.04*
**Heart failure**	146 (1.9)	2 (0.02)	11.7	2.2–60.4	0.03	1.9	1.7–114.3	0.02*
**Ischaemic heart disease**	32 (0.4)	6 (0.07)	1.6	0.3–7.7	0.60	2.2	0.3–14.1	0.40
**Peripheral arterial disease**	32 (0.4)	5 (0.06)	0.9	0.1–8.0	0.90	1.5	0.1–15.1	0.73
**Renal failure**	48 (0.6)	42 (0.5)	1.3	0.7–2.5	0.50	1.8	0.9–3.8	0.12
**Stroke**	22 (0.3)	22 (0.3)	0.0	0–0	0.99	0.0	0–0	0.99

Multivariable analysis adjusted for birthweight, congenital heart disease, gestation, SIMD, maternal antenatal smoking, maternal antenatal diabetes, and antenatal steroids.

CI, confidence interval; OR, odds ratio; SIMD, Scottish Index of Material Deprivation.

* *P* < 0.05.

## Discussion

Epidemiological, clinical, and experimental studies have implicated an important role for testosterone in cardiovascular (patho)physiology. Earlier investigations suggested that excess testosterone predisposed to CVD in men,^26^ while more recent studies implicate testosterone deficiency in premature coronary artery disease, atherosclerosis, and hypertension.^27,28^ These conflicting data likely relate to studies performed at different stages of development and the variable direct actions of testosterone on the cardiovascular system independent of effects on the reproductive system.

To address this and to explore putative molecular mechanisms underlying cardiovascular effects of testosterone deficiency, we studied hypospadias, as a marker of hypogonadism, throughout the lifespan from young boys with evidence of intrauterine androgen deficiency (short AGD) to adults.^29^ We used a multidisciplinary approach spanning molecular and vascular to epidemiological studies. Major findings from our study indicate that (i) adolescents with hypospadias have vascular structural changes evidenced by increased CIMT; (ii) in small arteries from young boys with hypospadias, endothelial function, and vascular reactivity are significantly impaired; and (iii) adult men with a history of hypospadias have increased risk of hospitalization with a primary diagnosis of heart failure, arrythmias, or hypertension (*Structured Graphical Abstract*).

We also identified Rho kinase activation, Nox5-induced ROS generation, redox-regulated signalling, and epigenetic changes in VSMCs as important molecular mechanisms underlying vascular alterations in hypospadias. Together our findings demonstrate that hypospadias predispose to functional and structural vascular alterations that may contribute to CVD in men. Low testosterone negatively impacts signalling in VSMCs by amplifying Rho kinase activation and inducing oxidative stress,^30,31^ processes that may contribute to vascular alterations in hypospadias, where there is inadequate androgen exposure during critical periods of foetal development. These novel findings identify hypospadias as an important risk factor in CVD in males, phenomena that are evident throughout the lifespan.

First, we undertook high-fidelity vascular phenotyping studies to determine if there were any clinical differences in vascular function associated with hypospadias. Children born with hypospadias had evidence of vascular remodelling (increased CIMT) and associated hypertension. These findings suggest that inadequate antenatal androgen exposure associated with hypospadias has pathophysiological effects that influence vascular function and promote blood pressure elevation. Children with untreated hypertension are at high risk of cardiovascular complications and target organ damage later in life.^32^ Treatment of paediatric hypertension reduces the risk of atherosclerosis in adulthood.^33^ Our finding of raised systolic blood pressure in adolescents born with hypospadias is important, because it suggests that even at this young age, hypospadias may exacerbate or augment risk factors that could lead to early CVD. Blood pressure is not routinely measured in boys who present clinically with hypospadias, but our results suggest that this may be important, especially since childhood hypertension is a risk factor for cardiovascular events in adulthood^34^ and testosterone deficiency is associated with premature CVD.^35,36^ Vascular phenotyping of adults born with hypospadias would be interesting to determine the progression of these findings.

Given the above findings, we next studied small arteries from boys with hypospadias. Vascular reactivity and endothelial function were assessed directly in isolated arteries. Small arteries are of interest, because they play an important role in total peripheral resistance and blood pressure regulation.^37^ We clearly show that studying small arteries from penile tissue from children is feasible and that these vessels can be examined by myography to directly assess vasoreactivity. Given that we also demonstrate that there is no difference in vascular function between arteries from skin and those from intra-abdominal fat, it is likely that these findings are true of all systemic resistance arteries and not specific to genital tissue. Of note, there was no correlation between the children’s blood pressure readings on the day of surgery and their vascular reactivity parameters. This may represent an insufficient sample size or that the vascular dysfunction demonstrated *in vivo* is subclinical at this age.

Our findings showed significant differences in vasoconstriction and vasodilation between boys with hypospadias and controls. Hypospadias was associated with impaired endothelial function, reduced vasorelaxation, and increased vasoreactivity, processes that were ameliorated by fasudil and NAC. These results indicate that abnormal vascular function likely involves Rho kinase- and ROS-dependent processes. Rho kinase activity regulates vascular smooth muscle function and increases MLC phosphorylation via inhibition of MLC phosphatase^38^ and has been implicated in development of cardiovascular disorders, as well as urogenital disorders including benign prostatic hyperplasia, erectile dysfunction, and prostate and bladder cancer.^39,40^ Reactive oxygen species are key intermediates in both normal physiological and pathological conditions and there are strong links between high levels of ROS and cardiovascular dysfunction and hypertension.^41^ Experimental models of androgen deficiency exhibit increased oxidative stress^42,43^ while clinical studies showed that infertile men and older males have reduced testosterone levels with associated increased production of ROS.^44,45^ Exact causes for oxidative stress in low testosterone conditions remain unclear but may relate to reduced *S*-nitrosoglutathione reductase activity, androgen receptor-independent processes, sirtuins, and altered antioxidant systems.^46,47^

To further delineate molecular mechanisms contributing to vascular pathology in low testosterone conditions, we studied primary culture VSMCs isolated from small arteries from boys born with hypospadias. Vascular smooth muscle cells from these boys exhibited significant perturbations in pro-contractile and pro-inflammatory signalling pathways. In particular, phosphorylation of MLC, a major signalling protein triggering VSMC contraction was increased in hypospadias. This was associated with upregulation of the RhoA/Rho kinase pathway (Rho kinase phosphorylation, Rho GEF expression) and increased expression of Ca^2+^ channels, without amplification of Ca^2+^ transients. Together these findings suggest that Rho kinase influences MLC sensitivity independently of Ca^2+^ changes, which may contribute to hypercontractility in hypospadias. The relationship between Rho kinase and testosterone deficiency has been demonstrated in experimental models of erectile dysfunction where Rho kinase inhibitors improved erectile function in castrated mice.^30,31^ Not all studies have shown an inverse relationship between testosterone and Rho kinase activity. In spontaneously hypertensive rats, Rho kinase amplified androgen-induced vasoconstriction.^40^

Associated with Rho kinase activation was increased NADPH-dependent generation of ROS and cellular oxidative stress. Of the many Nox isoforms identified in VSMCs,^48^ we found increased expression of Nox5 and hyperoxidation of redox-sensitive proteins (peroxiredoxin) in hypospadias. This was associated with decreased expression of Nox4, which has been described as a vasoprotective Nox isoform.^49,50^ When arteries from boys with hypospadias were incubated with melittin, a Nox5 inhibitor, or NAC, a ROS scavenger, hypercontractile responses were normalized. These findings are in keeping with the notion that Nox5-induced ROS production in VSMCs is involved in vascular alterations in low testosterone conditions. We previously described Nox5 as a pro-contractile Nox isoform,^24^ but our data here are the first to show Nox5 dysregulation in children. Associated with Nox5/ROS changes was peroxiredoxin hyperoxidation and DNA damage in VSMCs, processes associated with downregulation of antioxidant genes, further contributing to oxidative stress. We did not study the effects of mitochondrial ROS release in these VSMCs, which would be an interesting future direction.

Critical to the regulation of vascular tone is the balance between vasoconstriction and vasodilation. In our study, hypospadias was characterized by hypercontraction and reduced endothelium-dependent and -independent vasorelaxation. Fundamental to vasorelaxation is activation of NOS and NO generation. Nitric oxide is a critical cellular signalling molecule that maintains endothelial cell function and inhibits VSMC proliferation, migration, and inflammation.^51^ Reactive oxygen species produced by NADPH modulate NO levels. In our study, VSMCs expressed *eNOS*, *iNOS*, and *nNOS*. Boys with hypospadias had reduced *eNOS* and *iNOS* mRNA expression but increased nNOS expression. This pattern of NOS expression usually indicates cell stress.^52^ Reduced activation of the NOS/NO pathway typically leads to reduced vasorelaxation and increased vasoconstriction,^53^ consistent with the hypercontractility seen in arteries from boys with hypospadias. In our study, the combination of decreased NO together with increased ROS bioavailability amplifies the oxidative milieu further contributing to increased oxidative modification of proteins in hypospadias. These processes may also influence DNA regulation. Boys with hypospadias had increased DNMT activity compared with controls. DNA methylation is influenced by DNMTs, of which DNMT3A and DNMT3B regulate methylation during early development.^54^ Previous studies demonstrated increased *AR* gene methylation secondary to DNMT3A in the foreskin tissue of patients with hypospadias,^54^ suggesting epigenetic alterations, which may contribute to hypospadias and/or vascular abnormalities as we observed in our study. Investigation of differential methylation patterns warrants further research in future studies.

Finally, given the above findings, we wanted to determine whether men born with hypospadias had increased cardiovascular risk. To our knowledge, the findings here are the first to identify an association between hypospadias and increased susceptibility to arrythmias, heart failure, and hypertension. Previous studies demonstrated that hypogonadism is associated with long QT syndrome^55^ and torsades de pointes,^56^ and hypertension has been associated with low testosterone levels in men undergoing investigation for erectile dysfunction.^57^ Testosterone deficiency has also been linked to heart failure, with 25% of heart failure patients reported to have biochemical hypogonadism.^58^ As such, the increased susceptibility to these conditions may be a direct effect of inadequate testosterone exposure during a critical period of foetal programming.

Of course, these epidemiological data demonstrate correlation but not causation. Due to the nature of the data collection, it was not possible to account for any additional confounders, as these were not available as part of the routinely collected data. Many factors can influence future cardiovascular risk. In particular, lifestyle factors such as exercise and diet are known to result in increased CVD,^59^ which are not accounted for in this group, although the adolescents had similar levels of self-reported physical activity.

This work demonstrates a link between CVD and hypospadias and further studies are required to confirm these findings. It would be useful to confirm gene expression data with protein expression and to investigate *in vitro* findings regarding ROS generation *ex vivo*. In addition, an animal model may help to delineate the mechanisms of some of this work. Multiply passaged VSMCs may retain VSMC markers but not represent the same signalling pathways as primary myocytes, which may affect the mechanisms identified underlying the differences in vascular function. Ideally, endothelial cells would have been used to interrogate the influence of NO but we were not able to culture these successfully from the surgical specimens. Additional experiments including investigation of eNOS uncoupling would also be useful. *Table 1* also suggests a link between diseases resulting from fibrosis and hypospadias and a focus of future work could be on pro-fibrotic processes such as differences in the expression, deposition, and degradation of matrix components (collagens, fibronectin, and metalloproetases) by VSMCs.

Between 20 and 30% of boys with hypospadias will also have additional malformations,^12,60,61^ which can be extra-genital and include structural cardiac anomalies.^62^ In our study, there was phenotypic heterogeneity in the groups studied. However, no differences in vascular reactivity were identified between individuals with proximal hypospadias compared with those with distal hypospadias. Further work is required however to identify whether clinical correlates such as blood pressure or CIMT would differ with a less severe phenotype and also to identify whether any other clinical parameters such as parental smoking might affect cardiovascular risk in this cohort. Current European Society of Cardiology guidelines on hypertension advise that all adults aged >18 years should have regular blood pressure screening^63^ and in the light of the above findings, we suggest that this should be particularly emphasized for men born with hypospadias. Discussion with these individuals should also be considered regarding addressing any modifiable risk factors, such as body weight, exercise, diet, smoking, and alcohol consumption, in an attempt to reduce any potential increased cardiovascular risk.^64^

In conclusion, we show that boys with hypospadias have evidence of early vascular dysfunction including hypercontractility and impaired vasodilation secondary to increased Rho kinase activation and oxidative stress. This manifests in later life as vascular remodelling and raised systolic blood pressure in adolescence, with increased risk of admission to hospital for CVD in adulthood. Molecular mechanisms underlying these phenomena involve Nox5-induced ROS generation, Rho kinase activation, and downregulation of NOS/NO, processes that alter VSMC function. Exact processes whereby hypospadias result in these effects remain unclear but epigenetic changes, possibly *in utero*, may be important and warrant further investigation. Our findings indicate that hypospadias is associated with endothelial dysfunction and vascular injury early in life and that this predisposes to hypertension and cardiovascular events in adulthood. These novel findings not only delineate some molecular and vascular mechanisms but identify hypospadias as a novel cardiovascular risk factor in males. Prospective studies with longitudinal cardiovascular assessment are required to establish the clinical implications of the findings.

## Supplementary Material

ehac112_Supplementary_Data

## Declaration of Helsinki

This study complies with the Declaration of Helsinki. The study was approved by the locally appointed ethics committee and informed consent was obtained from the subjects.
