# Supplementary material for: Vascular dysfunction and increased cardiovascular risk in hypospadias
Source: Eur Heart J. 2022 Mar 17;43(19):1832–45. doi: 10.1093/eurheartj/ehac112 (PMC9113289; doi:10.1093/eurheartj/ehac112)
Supplement: ehac112_Supplementary_Data [file ehac112_supplementary_data.docx]

**Supplementary Material**

**Expanded Methods**

**Vascular phenotyping in adolescents**

*Ethics approval.* This study was authorised by the West of Scotland Research Ethics Committee 5 (REC reference: 17/WS/0063) and by the West of Scotland Research and Development Office (study reference GN17CA060P). The patient information sheets, example consent and assent forms and confirmation of ethics approval are available for this study at: <http://researchdata.gla.ac.uk/997/>.

*Recruitment.* Boys aged 12.0-18.9 years were recruited from endocrine or urology clinics at the Royal Hospital for Children, Glasgow. Written informed consent was obtained from the boy prior to participation. Cases were defined as boys born with hypospadias and controls were defined as boys with no previous history of hypospadias. Boys were excluded from recruitment if they had any associated comorbidity or were prescribed any medication. Clinical characteristics of the two groups are shown in Supplementary Table 1.

*Genetics.* All cases of proximal hypospadias within this study had undergone the local targeted gene panel for Disorders of Sex Development as previously described (1).

*Blood pressure*. Blood pressure was recorded using a Welch Allyn DS55 (Welch Allyn, UK) sphygmometer. It was taken in the right arm with the boy seated, and the arm supported on a table. The blood pressure cuff varied according to the size of the boy, with the width of the cuff covering at least 75% of the upper arm from the acromion to the olecranon **(2)**. Prior to blood pressure measurement, the boys were asked to sit quietly for a minimum of 5 minutes. and a mean of 3 blood pressure readings was taken as per current guidance **(2, 3)**. Blood pressure SDS were calculated according to gender, height and age **(2)**.

*Carotid intima media thickness (CIMT).* CIMT measurements were obtained with the Acuson Sequoia C512 (Siemens, Germany) ultrasound machine, using the 9.0 MHz transducer probe. The participant was asked to lie flat with his head turned to 90º. Ultrasound gel was applied and a longitudinal view of the common carotid artery was taken, 1-2cm from the carotid bulb **(4, 5)**. The mean of ten consecutive CIMT measurements was obtained and the procedure was repeated on the contralateral side and a mean value for both sides was calculated. CIMT SDS were calculated using reference data from Doyon et al. **(5)**.

*Flow mediated dilatation (FMD).* FMD was measured whilst the patient was fasting using the UNEX EF (UNEX Corporation, Japan) machine. This is a semi-automated device which uses B-Mode ultrasound to simultaneously capture longitudinal and cross-sectional views of the brachial artery (6). Participants were asked to lie supine with their right arm outstretched on the machine apparatus for 5 minutes in a quiet temperature-controlled room for 5 minutes. During this time an occlusion cuff was placed on the right forearm and two ECG cuffs were attached to each wrist. Thereafter, a brachial cuff was placed on the left arm to obtain a resting blood pressure. This was used to determine how much occlusion was required for analysis, with the occlusion cuff being inflated 50mmHg above systolic blood pressure. The ultrasound probe was placed approximately 5cm proximal to the elbow and secured using a probe holder, once an adequate image of the brachial artery was obtained. The UNEX EF software calculated the brachial artery’s rest diameter from the intima-intima via a tracking gate. The occlusion cuff was then inflated for 5 minutes. During this time, the participant was reminded not to move their head or body, despite any sensations of paraesthesia. Afterwards the cuff was deflated and the brachial artery was tracked by the software for 2 minutes. The diameter of the brachial artery per beat was synchronised with the R waves measured by the ECG cuffs. Automated outputs for rest diameter, maximum diameter and % FMD (as measured automatically by [maximum diameter-rest diameter)/rest diameter]*100) were recorded. Nitroglycerine-induced vasodilation was not used as a control, as this would not be standard practice in FMD in children.

*Pulse wave velocity**.* Carotid femoral PWV was measured using the SphygmoCor XCEL (Atcor Medical, Australia). A femoral cuff was placed around the right thigh of the participant, who was asked to lie supine. The right carotid artery was then palpated and the distances between the location of the strongest pulse point to the jugular notch and between the jugular notch and the top of the femoral cuff were measured and inputted into the SphygmoCor XCEL software. The carotid pulse was then acquired using applanation tonometry and the femoral pulse was measured by assessing the volumetric displacement of the cuff **(7)** and the carotid femoral pulse wave velocity reported by the SphygmoCor XCEL software. PWV SDS were calculated according to age and height using reference data from Reusz et al **(8)**. Where there was a discrepancy between the SDS for age or height, the highest centile was used.

### *Pulse wave analysis (PWA)*. Pulse wave analysis was undertaken using the SphygmoCor XCEL (Atcor Medical, Australia) device. A brachial cuff, sized as per manufacturer’s instructions, was applied as the participant was lying supine. The SphygmoCor XCEL software then generates a brachial pressure waveform, whilst measuring brachial systolic and diastolic blood pressure and this is transformed into central aortic waveforms automatically (9).

*Questionnaires*. To adjust for physical fitness and health-related quality of life (HRQoL) which may affect vascular status, all participants were asked to complete the KIDSREEN 52 questionnaire for self-perceived quality of life and the Physical Activity Questionnaire for Adolescents (PAQA) was used to assess physical activity.

**Serum and urine analysis**

Where possible, bloods were taken from all boys for plasma testosterone, anti-Müllerian hormone, FSH and LH levels as well as fasting cholesterol, triglycerides and glucose. In boys with hypospadias, a further 1ml of blood was stored for genetic analysis, where appropriate, which was undertaken in the local NHS genetics laboratory. This analysis was undertaken in the local NHS laboratory as per standard protocols. Serum and urine were analysed for the following:

*Total Antioxidant Capacity (TAOC).* TAOC was measured by the conversion of Cu^2+^ ions to Cu^2+^ by small molecule antioxidants using a commercial kit as per manufacturer’s instructions (Abcam, UK). Results are expressed as % control.

*8-hydroxy 2 deoxyguanosine (8 OH-dG) levels.* 8 OH-dG was measured using a commercial kit according to the manufacturer’s ELISA kit instructions (Abcam, UK). Results are expressed as % control.

*Thiobarbituric Acid Reactive Substances (TBARS)*. TBARS were measured in plasma to assess lipid peroxidation using a commercial kit as per manufacturer’s instructions (Caymen Chemical, USA). Results are expressed as % control.

**Vascular reactivity in young boys**

*Ethics approval.* This study was authorised by the West of Scotland Research Ethics Committee (REC reference: 16/WS/0186) and by the West of Scotland Research and Development Office (study reference GN16CA519). The patient information sheets, example consent and assent forms and confirmation of ethics approval are available for this study at: [***http://researchdata.gla.ac.uk/998/***](http://researchdata.gla.ac.uk/998/).

*Recruitment*. Excess foreskin tissue was obtained from boys undergoing routine urological surgery at the Royal Hospital for Children, Glasgow. Written informed consent was obtained from the parents of each boy prior to surgery. Written assent or consent was also obtained from the boy himself, where appropriate depending on the developmental stage of the boy. Cases were defined as boys undergoing hypospadias repair and controls were defined as boys undergoing circumcision. Samples were obtained from the same area of the foreskin in both groups. Boys were excluded from recruitment if they had any associated other comorbidity, such as a hormone deficiency or other chronic medical problem, or were prescribed any medication, such as current use of antibiotics, immunosuppressants, analgesia, antidepressants, or stimulants or which may alter their vascular status. Clinical characteristics of the 2 groups are shown in Supplementary Table 2.

*Wire myography*. Subcutaneous arteries were dissected from the skin and 1.5-2mm lengths of arteries were mounted onto wire myographs (AD Instruments, UK) for vascular reactivity studies. After mounting, vessels were normalised after 30 minutes of rest and then washed with PSS (0.25M NaCl, 0.001M KCl, 50mM NAHC0_3_, 2 mM KH_2_PO_4_, 1 mM glucose, 2.5 mM CaCl_2_). Functionality of the vessels was assessed by contraction to 62.5 mM of KPSS (PSS with 62.5mM KCl) on 2 occasions. Constriction curves were then conducted using cumulative increasing doses of the thromboxane A2 analogue U46619 (Enzo Life Sciences, UK) (1x10^-10^-3x10^-6^). Endothelium dependent relaxation was assessed using relaxation curves to acetylcholine (ACh) (Sigma Aldrich, UK) (1x10^-9^-3x10^-5^) and endothelium independent vasodilation was assessed using sodium nitroprusside (SNP) (Honeywell, UK) (1x10^-9^-3x10^-5^). After each curve, vessels were washed 3 times with PSS prior to further incubation. Where the same condition was used more than once in the same patient, an average of the results was used for analysis.

**Molecular studies in primary culture vascular smooth muscle cells (VSMC)**

*Culture of VSMC culture from small arteries from young boys.* Small arteries from the penile skin were cleaned of connective tissue and pooled in a sterile Eppendorf tube. VSMCs were dissociated using an enzymatic digestion mix at 37°C using gentle agitation. The cell suspension was then centrifuged and resuspended in complete F12 medium (Sigma Aldrich, UK). Within 48 hours the medium was replaced with complete VSMC medium (231 medium (Invitrogen, UK); 25ml SMGS (Invitrogen, UK); 5ml penicillin/streptomycin). The cells were then grown until confluence in flasks and split using trypsin until passages 4-6. Once confluent, VSMCs were rendered quiescent with serum deprivation (5% SMGS in 500ml VSMC medium 231) overnight and then incubated with either drugs or 5µl vehicle depending on the specific protocol used. Smooth muscle cell origin of the cells was confirmed by assessment of protein expression of markers for VSMCs and fibroblasts. Controls were unstimulated. After stimulation plates were washed with ice cold non-sterile PBS and stored in -20°C until use.

*Quantitative real-time polymerase chain reaction (qRT-PCR).* mRNA expression was measured on VSMCs using quantitative real-time polymerase chain reaction. Total RNA was extracted using Qiazol (Qiagen, UK). Target gene expression was identified using Qiagen QuantiTech primer assays (Qiagen, UK) and SYBR^®^ Green (UK). Primers used are shown in Supplementary Table 1. Transcript gene expression was normalised using the housekeeping gene. The 2^δδCT^ method was then used to calculate relative gene expression (10). Results are expressed as % control. Primers used are detailed in Supplementary Table 3.

*Immunoblotting.* VSMC proteins were extracted and separated by electrophoresis on pre-casted 4-20% tris-glycine polyacrylamide gels (Invitrogen, UK). These were then transferred to a nitrocellulose membrane and blocked in 5% bovine serum album in tris-buffered saline solution. Membranes were incubated with primary antibodies overnight at 4°C. Thereafter they were washed 3x in TBS containing 0.1% Tween. Membranes were then incubated with secondary antibodies for 1 hour at room temperature and fluorescent signals measured using the Odyssey CLX, LI-COR® scanner and analysed using ImageStudioLite software. Alpha tubulin (1:10,000, 3% BSA/TBST) was used on all membranes as an internal housekeeping loading control protein. Results were normalised according to cellular protein levels. Results are expressed as % control. Antibodies used are detailed in Supplementary Table 4.

*Calcium signaling*. Fluorescent measurement of the Ca^2+^ indicator Cal-520 acetoxymthyl ester (Cal-520/AM, Abcam, 10µmol/L) in response to the presence or absence of ionomycin (1µmol/L) was used to identify differences in Ca^2+^ in VSMCs from boys with hypospadias and controls using an inverted epifluorescence microscope (Zeiss, UK) with excitatory wavelengths of 490nm and emission at 535nm.

*Lucigenin enhanced chemiluminescence*. VSMCs were washed in lysis buffer (20mM KH_2_PO_4_, 1mM EGTA, 10µg aprotinin, 10 µg leupeptin, 10µg pepstatin, 1mM PMSF). Lucigenin (5µmol/L) (Sigma-Aldrich, UK) was added and basal luminescence measured using a luminometer (Orion II, Titerek-Berthold, Germany). NADPH (10^-4^) (Calbiochem®, UK) was then added and measurements repeated. Results were normalised according to the protein concentration of the cell lysate as measured using the Bradford method via the Spectra Max M2 (Molecular Devices, USA) machine and Softmax (Molecular Devices, USA) software. Results are expressed as % control in relative light units (RLU) per microgram of protein.

*Electron Paramagnetic Resonance (EPR)*. VSMCs were incubated in the dark at 37°C for 30 minutes with 10 mM of 1-hydroxy-3-carboxy-2,2,5,5-tetramethylpyrrolidine (CPH) spin probe (Noxygen, Germany). EPR spectra and kinetics were recorded using an EPR spectrometer (e-scan R Bruker Biospin GmbH, Germany) at the following settings: 3375G centre field; 2.27G modulation amplitude; 5.24 second sweep time; 60G sweep width; 10 scans. The concentration of CPH was determined from the amplitude of the low field component of EPR spectra. Results are expressed in µmol/minute.

*Amplex red*. H_2_O_2_ levels were determined in cell lysates using a fluorescence Amplex Red assay kit as per manufacturer’s instructions (Molecular Probes, UK). Results were normalised according to cellular protein levels, as measured by the Bradford assay. Results ae expressed as % control in units per milligram protein.

*Rho kinase*. Rho kinase levels were assessed in cell lysates using a commercial Rho kinase activity kit as per manufacturer’s instructions (Cell Biolabs, UK).

*Detection of nitric oxide (NO).* Nitric oxide was measured in cell lysates after 30 minutes of incubation with 0.3mM DAF-FM diacetate (4-Amino-5-Methylamino-2',7'-Difluorofluorescein Diacetate) probe (Thermo Fisher, UK). Fluorescence was measured at an excitation of 495nm and emission of 515nm using the Spectra Max M2 (Molecular Devices, USA) machine and Softmax (Molecular Devices, USA) software. Results are normalised per microgram of protein.

*Peroxynitrite detection*. Peroxynitrite levels in VSMC lysates were measured using a commercial kit according to manufacturer’s instructions (Abcam UK). Results were normalised according to cellular protein levels.

*DNA Methyltransferase (DNMT) activity*. DNMT activity was determined in VSMCs using a commercial kit according to the manufacturers’ ELISA kit instructions (Abcam, UK). Results were normalised according to cellular protein levels. Results are expressed as % control.

Drugs: The following drugs and doses were used in the vascular experiments: melittin (10^-7^M, Sigma Aldrich, UK), L-NAME (10^-4^M, Sigma Aldrich, UK), NAC (10^-5^M, Sigma Aldrich, UK) and ICI192,605 (10^-8^M, Tocris, UK). Drugs were incubated for 30 minutes.

**Data linkage studies**

In Scotland, data on all National Health Service encounters has been routinely collected since January 1980, using the Scottish Morbidity Record (SMR) Scheme. At the time of presentation or medication prescription, hospital software is used to code the underlying diagnosis using the World Health Organisation International Classification of Diseases (ICD-9 before 1996 and ICD-10 after 1996). Ethics approval was sought from the National Health Service (NHS) Information Services Division (ISD) Public Benefit and Privacy Panel for Health and Social Care (PBPP) (reference number 1718-0177). Pseudo-anonymised data were then obtained from ISD on all admissions to hospital secondary to a cardiometabolic diagnosis (arrhythmia, angina, cardiomyopathy, diabetes, hypertension, heart failure, ischemic heart disease, myocardial infarction, peripheral arterial disease, renal failure, stroke) for men in the database with the ICD-10 code of Q54 for hypospadias and for male, age, gestation, birthweight and Scottish Index of Multiple Deprivation (SIMD) matched controls. No pre-specified sample size calculation was used as we wished to obtain all available data.

The SIMD is an index of deprivation that takes into account data on employment, average income, health, education, housing, crime and access to local services. The lower the number, the more socially deprived the geographical region. Data were matched by eDRIS (Public Health Scotland) by age, birthweight, gestation and postcode sector at a ratio of 1:1 where possible. Additional controls were provided to account for other conditions also requested at the time of data release but not pertinent to this study. Data were available from January 1980 until May 2019 (earliest time point available until time of data linkage by ISD). A total of 1, 300, 399 case records were obtained regarding 35, 942 individuals. Of these, 18, 050 had an ICD-10 diagnosis of hypospadias (Q54). Only individuals over the age of 18 years old at the time of December 2019 were included in analysis, giving a final cohort of 7, 591 men with hypospadias. Data on all were received using the Scottish Morbidity Record 01: Inpatient records (SMR01) from 1981 (earliest data available until the date of data receipt in May 2019).

To minimise confounding variables, data were also sought on the following routinely collected variables: gestational age at birth; birthweight; maternal smoking in pregnancy; SIMD score at birth; antenatal steroids; presence of congenital heart disease; and maternal diabetes in pregnancy from the Scottish Birth Record and the Scottish Morbidity Record 02: maternity records (SMR02). Not all data were available for all individuals and this was corrected via imputation. Data regarding other confounding factors such as current smoking status were not available, due to the nature of the ISD database. Men were excluded only if they were <18 years of age at the time of data analysis or if they had a previous history of congenital heart disease. Clinical characteristics of the 2 groups are shown in Supplementary Table 5.

**Statistical analysis**

*Vascular phenotyping*. GraphPad Prism 8.0 software (GraphPad Software Inc, USA) was used for all statistical analysis. The Shapiro-Wilks normality test was performed for all data to determine if data were parametric or not. Differences between groups were calculated using 1-way ANOVA followed by Tukey’s post-test to correct for multiple testing or Student’s t tests when appropriate. A p value of <0.05 was deemed statistically significant. Multiple linear regression was used to compare correlations between groups using the sum of least squares model.

*Vascular reactivity and molecular studies.* Mean ± SEM were calculated for all data. Normality of all data was assessed using the Shapiro-Wilks test and statistical outliers removed from groups after review using the ROUT method. Differences between groups were calculated using 1-way ANOVA followed by Tukey’s post test, Student’s t tests or Wilcoxon signed-rank tests when appropriate. Linear regression and Pearson correlation coefficients were calculated to assess the relationships between groups. For all analyses, a p value of <0.05 was deemed statistically significant. All statistical analysis was performed using GraphPad Prism 8.0 software (GraphPad Software Inc, USA).

*Data linkage*. Data from each database was linked using R version R3.6.3 (R Foundation for Statistical Computing, USA). Analysis was performed using SPSS version 22.0 (IBM, USA). Categorical variables were compared between groups using chi square analysis and are shown as counts and percentages. Continuous variables were compared using independent t tests and are shown as median (range). Regression models were constructed to adjust for all of the potential confounding variables requested, as shown in Table 1. Fishers exact test was used to determine predictors of cardiovascular admission for all admissions, as shown in Supplementary Table 6.

**Supplementary Results**

*Optimising myography to assess vascular function in small arteries from penile skin from children.*

To our knowledge these are the first studies to examine, by myography, subcutaneous small arteries from penile skin from children and accordingly we needed to optimise conditions taking various factors into consideration including: i) type of regional anesthetic used; ii) whether penile skin small arteries are representative of systemic peripheral resistance arteries; iii) most appropriate vasoconstrictor to assess vasoconstriction; and iv) influence of ethnic background, birthweight or age.

Anesthetic agents and approaches can differentially influence vascular function (vasoconstriction/vasodilation) (11, 12). In all children, anaesthesia was induced with isoflurane and the regional anesthetic injected was always bupivacaine but depending on the approach by different surgeons this was injected as a caudal anesthetic block or via penile block. Where arteries were obtained from surgeries with a penile block, vascular contraction assessed by myography was significantly reduced compared to arteries from surgeries with a caudal block (Emax % KCl: 42.4 *vs* 72.1, p=0.04) (Supplementary Figure 1A). In light of these data and due to the fact that 23/27 (85%) of hypospadias surgeries recruited to this surgery were undertaken using caudal anaesthesia, vascular reactivity data obtained from surgeries using only caudal anaesthesia are reported in this study.

To ensure that the data generated from arteries from penile skin recapitulate vascular reactivity from other peripheral vessels, comparisons were made with arteries from intra-abdominal fat obtained during orchidopexy from 4 healthy controls with unilateral undescended testis and 4 boys with hypospadias with unilateral undescended testis (Supplementary Figure 1B), demonstrating no difference in contraction between vessels from different vascular regions.

Initial experiments also sought to identify the most appropriate vasoconstrictor to use in myography studies. Contraction curves were performed using U46619, serotonin (5-HT), noradrenaline (NA) and phenylephrine (1x10^-10^-3x10^-5^). The response to U46619 was most consistent and this was, therefore, chosen as the optimal vasoconstrictor for further studies (Supplementary Figure 1C).

Given that 85% of the circumcisions were performed in boys of non-Caucasian descent compared to 38% of boys with hypospadias, analysis was undertaken to determine if there was any difference in contraction depending on ethnicity of the subjects. No statistically significant difference in contraction was observed between Caucasian and non-Caucasian control patients (Emax % KCl:62.4 *vs* 74.7, p=0.4) (Supplementary Figure 2A). Analysis of patient birthweight SDS also demonstrated no differences in the number of arteries obtained, maximal contraction to KPSS or contraction to U46619 (Supplementary Figure 2B).

In addition, analysis was undertaken to determine if the age of the patient affected the number of arteries obtained, maximal contraction to KPSS or contraction to U46619, with no differences seen in those under the age of 2 years compared to children who were 3 years old or older (Supplementary Figure 2C).

| Median (range) | Boys with hypospadias  n=14 | Controls  n=14 | p |
| --- | --- | --- | --- |
| Clinical characteristics |  |  |  |
| Age (years) | 14.3 (12, 17.2) | 14.6 (12.1, 18.5) | 0.6 |
| Gestation at birth (weeks) | 40 (33, 41) | 38 (32, 42) | 0.2 |
| Birthweight (kg) | 3.3 (1.0, 3.8) | 3.1 (0.8, 3.7) | 0.6 |
| Birthweight SDS |  |  |  |
| Height SDS | -0.3 (-1.5, 2.2) | -1.1 (-3.1, 1.2) | 0.6 |
| Weight SDS | -0.1 (-2.5, 2.0) | -0.7 (-3, 2) | 0.7 |
| EMS | 8 (3, 11) | 12 (12, 12) | <0.0001 |
| Tanner stage | III (I, IV) | II (I, IV) | 0.3 |
| Position of hypospadias  (P, M, D) | 14, 0, 0 | - | - |
| Fasting blood hormone profile |  |  |  |
| LH (U/l) | 3.2 (0.8, 12.1) | 1.2 (0.8, 2.3) | *0.03 |
| FSH (U/l) | 7.8 (1.3, 46.1) | 1.9 (0.6, 7) | ***0.003 |
| Testosterone (nmol/l) | 10.9 (4, 39.4) | 4.1 (<0.5, 22.1) | 0.1 |
| AMH (pmol/l) | 45.5 (18, 118) | 223 (26-352) | 0.02 |
| Oestradiol (mmol/l) | <70 (<70, <70) | <70 (<70, <70) | >0.99 |
| Cholesterol (mmol/l) | 3.8 (2.9, 4.9) | 4 .3 (2.2, 4.6) | 0.9 |
| Triglycerides (mmol/l) | 0.7 (0.5, 1.4) | 0.7 (0.4, 0.8) | 0.2 |
| Fasting glucose (mmol/l) | 4.8 (4-5.4) | 4.4 (4-4.7) | 0.09 |

Supplementary Table 1. Clinical characteristics and fasting blood levels of adolescents in the vascular phenotyping study. Data shown as median (range). Abbreviations: AMH: anti-Müllerian hormone; D: distal; EMS: external masculinisation score; FSH: Follicle Stimulating Hormone; LH: Luteinising Hormone; M: mid shaft; P: proximal; SDS: standard deviation score. *p<0.05, ****p<0.0001.

| Median (range) | Boys with hypospadias  n=27 | Controls  n=37 | p |
| --- | --- | --- | --- |
| Clinical characteristics |  |  |  |
| Age (years) | 1.9 (0.8, 6.4) | 2.0 (1.2, 12.9) | 0.23 |
| Gestation at birth (weeks) | 40 (33, 41) | 40 (36, 42) | 0.15 |
| Birthweight (kg) | 3.3 (0.7, 4.0) | 3.5 (1.5, 4.6) | 0.28 |
| Birthweight SDS | -0.8 (-2.9, 0.6) | -0.9 (-1.3, 0.5) | 0.9 |
| Height SDS | 0.1 (-2.5, 3.1) | -0.3 (-2.5, 2) | 0.6 |
| Weight SDS | 0.5 (-2.5, 2.0) | 0.3 (-3, 2) | 0.8 |
| EMS | 8 (3, 11) | 12 (12, 12) | **** <0.0001 |
| Tanner stage | - | - | - |
| Position of hypospadias  (P, M, D) | 8, 6, 13 | - | - |
| Penile length (mm) | 28.1 (20, 50) | - | - |
| Anogenital distance (mm) | 30.4 (22.4, 39) | - | - |
| Anoscrotal distance (mm) | 23.5 (13.7, 36.4) |  |  |
| Fasting blood hormone profile |  |  |  |
| LH (U/l) | <0.1 (<0.1, 1.4) | <0.1 (<0.1, 0.5) | 0.92 |
| FSH (U/l) | 0.7 (<0.1, 3.0) | 0.8 (0.2, 1.5) | 0.61 |
| Testosterone (nmol/l) | <0.5 (<0.5, <0.5) | <0.5 (<0.5, <0.5) | >0.99 |
| AMH (pmol/l) | 875 (421, 1472) | 987 (371, 1725) | 0.98 |
| Cholesterol (mmol/l) | 4.4 (3.2, 4.9) | 4 (2, 4.7) | 0.29 |
| Triglycerides (mmol/l) | 1.1 (0.7, 1.8) | 0.8 (0.6, 1.7) | 0.04 |
| Oestradiol (mmol/l) | <70 (<70, <70) | <70 (<70, <70) | >0.99 |
| Fasting glucose (mmol/l) | 4.3 (3.4, 5.3) | 4.7 (3.5, 6.8) | 0.38 |
| Sample blood vessel characteristics |  |  |  |
| Diameter of arteries (microns) | 232 (170, 316) | 267 (200, 595) | 0.02 |
| Number of arteries dissected | 4 (0, 6) | 6 (0-8) | **** <0.0001 |

Supplementary Table 2. Clinical characteristics and fasting blood levels of young participants undergoing hypospadias repair or circumcision. Data shown as median (range). Abbreviations: AMH: anti-Müllerian hormone; D: distal; EMS: external masculinisation score ; FSH: Follicle Stimulating Hormone; LH: Luteinising Hormone; M: mid shaft; P: proximal; SDS: standard deviation score. ****p<0.0001.

| Gene | Forward Primer | Reverse Primer |
| --- | --- | --- |
| L-type voltage-dependent calcium channel (CACNA1C) | 5’ – GAA GCG GCA GCA ATA TGG GA – 3’ | 5’ – TTG GTG GCG TTG GAA TCA TCT – 3’ |
| Endothelial NOS (eNOS) | 5’ - ACC CTC ACC GCT ACA ACA TC - 3’ | 5’- GCT CAT TCT CCA GGT GCT TC-3’ |
| Glyceraldehyde 3-phosphate dehydrogenase (GAPDH) | 5’ - GAG TCA ACG GAT TTG GTC GT – 3’ | 5’- TTG ATT TTG GAG GGA TCT CG – 3’ |
| Inducible NOS (iNOS) | 5’- ACA AGC CTA CCC CTC CAG AT – 3’ | 5’ - TCC CGT CAG TTG GTA GGT TC – 3’ |
| Inositol 1,4,5-triphosphate receptor (IP3R) | 5’ – GCG GAG GGA TCG ACA AAT GG – 3’ | 5’ – TGG GAC ATA GCT TAA AGA GGC A – 3’ |
| Neuronal NOS (nNOS) | 5’ – GTG AAG CTG GAG TGC TTT CG – 3’ | 5’ – CCT GAC TCT CGG AAC TTG ATG AG – 3’ |
| NADPH oxidase 1 (Nox1) | 5’ – TCA CCA ATT CCC AGG ATT GA- 3’ | 5’ – TGT GGT CTG CAC ACT GGA AT-3 ’ |
| NADPH oxidase 2 (Nox2) | 5’ – GTC ACA CCC TTC ATC CAT TCT CAA GTC AGT- 3’ | 5’ – CTG AGA CTC ATC CCA GCC AGT GAG GTAG- 3’ |
| NADPH oxidase 4 (Nox4) | 5’ – TGC AGC AAG ATA CCG AGA TG- 3’ | 5’ – GTG ATC ATG AGG AAT AGC AC- 3’ |
| NADPH oxidase 5 (Nox5) | 5’ – GCA GGA GAA GAT GGG GAG AT- 3’ | 5’ – CGG AGT AGG GCA AAG- 3’ |
| RhoGEF LARG | 5’ – ACA CAG TCT ACT ATC ACC GAC A – 3’ | 5’ – TGC AAT GCG CTC AAC TTT CTG – 3’ |
| RhoGEF PDZ | 5’ – ATG AGT GTA AGG TTA CCC CAG AG – 3’ | 5’ – CGT TGA ACG AGA CCT GTT GT – 3’ |
| RhoGEF P115 | 5’ – GAT GCA ACA TAC CAT CTC TAC CG – 3’ | 5’ – CCC CGA CTT CTT GTC TCC AC – 3’ |
| Ryanodine receptor 1 (RyR1) | 5’ – CTC CGC CTC TTT CAT GGA CAT – 3’ | 5’ – CTG CCC GGT AGT GAC ATG C – 3’ |
| Ryanodine receptor 2 (RyR2) | 5’ – ACA ACA GAA GCT ATG CTT GGC – 3’ | 5’ – GAG GAG TGT TCG ATG ACC ACC – 3’ |
| Ryanodine receptor 3 (RyR3) | 5’ – GGA CTT GGG AAT CGC CTG TG – 3’ | 5’ – GCT CTG ACA GAT AGG GAC TGT TC – 3’ |
| Sarcoplasmic/Endoplasmic Reticulum Calcium ATPases (SERCA) | 5’ – AAA CCA CGG AGG AAT GTT TGG – 3’ | 5’ – AGC TCA TTG AGG CCG TAT TTC – 3’ |
| Sodium dismutase 1 (SOD1) | 5’- GAA GGT GTG GGG AAG CAT TA -3’ | 5’- ACA TTG CCC AAG TCT CCA AC – 3’ |
| Thromboxane A2 receptor (TBXA2R) | 5’ – GCT ACC TGG GTA TCA CCC G – 3’ | 5’ – CAC GCG CAA GTA GAT GAG CA – 3’ |
| Transient receptor potential cation channel, subfamily M, member 2 (TRPM2) | 5’ – TTC GTG GAT TCC TGA AAA CAT CA – 3’ | 5’ – CCA GCA TCA GAC AGT TTG GAA C – 3’ |

Supplementary Table 4. Primers used for mRNA expression studies.

| Name | Manufacturer | Dilution | Host species |
| --- | --- | --- | --- |
| Alpha tubulin | Abcam, UK | 1:10 000 | Rabbit |
| pMLC20 | Cell Signalling, UK | 1:1000 | Rabbit |
| Prx-SO3 | Abcam, UK | 1:10 000 | Rabbit |
| Anti-rabbit Alexa Fluor 488 | Life Technologies | 1:10 000 | Goat |

Supplementary Table 4. Primary and secondary antibodies used for immunoblotting experiments.

|  | Controls  n=8,073 | Men with hypospadias  n=6,797 | p |
| --- | --- | --- | --- |
| Birthweight (kg) | 3.3 (0.6, 5.4) | 3.3 (0.6, 5.8) | 0.07 |
| Gestation (weeks) | 39 (24, 44) | 39 (24, 42) | 0.1 |
| Age (years) | 29 (18, 38) | 23 (18, 38) | 0.2 |
| Maternal smokers | 1104 (13.6%) | 388 (5.7%) | 0.09 |
| SIMD | 4 (0, 10) | 6 (0, 10) | 0.08 |
| Full course AN steroids | 29 (0.4%) | 28 (0.4%) | 0.1 |
| Incomplete course AN steroids | 707 (8.8%) | 368 (5.4%) | 0.0001 |
| Maternal diabetes | 469 (5.8%) | 243 (3.6%) | 0.4 |

Supplementary Table 5. Clinical characteristics of men included in data linkage studies.

Abbreviations: AN: antenatal; SIMD: Scottish Index of Material Deprivation.

| Admission diagnosis | No of admissions men with hypospadias (%) | No of admissions controls (%) | p |
| --- | --- | --- | --- |
| Arrhythmia | 36 (0.5) | 16 (0.2) | *0.0007 |
| Angina | 1 (0.01) | 1 (0.01) | >0.9 |
| Cardiomyopathy | 16 (0.2) | 4 (0.05) | *0.0026 |
| Diabetes | 71 (0.9) | 65 (0.8) | 0.14 |
| Hypertension | 51 (0.7) | 14 (0.2) | *<0.0001 |
| Heart failure | 146 (1.9) | 2 (0.02) | *0.0007 |
| Ischaemic heart disease | 32 (0.4) | 6 (0.07) | *<0.0001 |
| Myocardial infarction | 7 (0.09) | 3 (0.03) | 0.20 |
| Peripheral arterial disease | 32 (0.4) | 5 (0.06) | *<0.0001 |
| Renal failure | 48 (0.6) | 42 (0.5) | 0.17 |
| Stroke | 22 (0.3) | 22 (0.3) | *0.0012 |

Supplementary Table 6. Frequency of admission for cardiometabolic diseases in 6, 797 men with hypospadias compared to 8,073 controls. Analysis via Fisher Exact test. *p<0.05.

Supplementary Figure 1. Optimization of myography technique in control arteries. A - Vascular contraction was significantly reduced in arteries obtained post penile block compared to arteries from surgeries with a caudal block. B - No differences in maximal contraction were seen between arteries from subcutaneous skin samples or abdominal fat samples post orchidopexy, although the reactions from penile skins were more variable, as demonstrated by larger confidence intervals. C - Arteries constricted most in response to 5-HT (Emax:90.5) and least in response to phenylephrine (Emax:32.3). The contractile response to U46619 was most consistent. Results are mean ± 95% CI of 8-9 arteries per group. Best fit cumulative concentration curves were compared with the extra sum-of-squares *F* test. Abbreviations: 5-HT: serotonin; KCl: potassium chloride; NA: noradrenaline; Phe: phenylephrine.

Supplementary Figure 2. Characteristics of isolated arteries. A - No statistically significant difference in contraction was seen between Caucasian and non-Caucasian control patients. B – No statistically significant difference in contraction in response to U46619 was seen in response to patient birthweight SDS. C – There was no difference in contraction to U46619 in those under the age of 2 years compared to children who were 3 years old or older. Best fit cumulative concentration curves were compared with the extra sum-of-squares *F* test. Results are mean ± SD of 6-9 arteries per group. Abbreviations: BW: birthweight; KCl: potassium chloride; SDS: standard deviation score.

Supplementary Figure 3. Vascular reactivity of arteries from boys with hypospadias and controls. A - Vessels from boys with hypospadias (grey circle symbols and line) demonstrated hypercontractility (Emax) compared to vessels from controls (black square symbols and line) (A). Compared to arteries from controls, arteries from cases had reduced endothelium-dependent (C) and endothelium-independent vasorelaxation (D). Results are mean ± SD CI of blood vessels from 27 boys with hypospadias and 37 controls. Where more than 1 blood vessel was obtained from the same boy, the mean was used. Best fit cumulative concentration curves were compared with the extra sum-of-squares *F* test. Abbreviations: KCl: potassium chloride; Ach: acetylcholine; SNP: sodium nitroprusside.

Supplementary Figure 4. TBXA2R expression. There was no significant difference between *TBXA2R* mRNA receptor expression in boys with hypospadias versus controls. Results are shown as % control from 18 controls and 15 boys with hypospadias, p=0.46. Data were analysed by Wilcoxon signed-rank test. Abbreviation: *TBXA2R*: thromboxane A2 receptor.

Supplementary Figure 5. mRNA expression of *iNOS*, *eNOS* and *nNOS*. VSMCs from boys with hypospadias have reduced *iNOS* (A) and *eNOS* (B) compared to controls but increased *nNOS* expression) (C). Abbreviations: *eNOS*: endothelial nitric oxide synthase; *iNOS*: inducible nitric oxide synthase; *nNOS*: neuronal nitric oxide synthase. Data were analysed by Wilcoxon signed-rank test.

Supplementary Figure 6. SOD1 expression. VSMCs from boys with hypospadias had reduced expression of *SOD1* compared to controls. Results are shown as % control from 18 controls and 15 cases. Data were analysed by Wilcoxon signed-rank test.

Supplementary Figure 7. Effects of L-NAME on superoxide production. VSMCs from boys with hypospadias had reduced superoxide production when incubated with L-NAME, a NO synthase inhibitor for 5 minutes or 2 hours. Results are shown as mean ± 95% CI from 6-15 cases. Data were analysed by Wilcoxon signed-rank test.


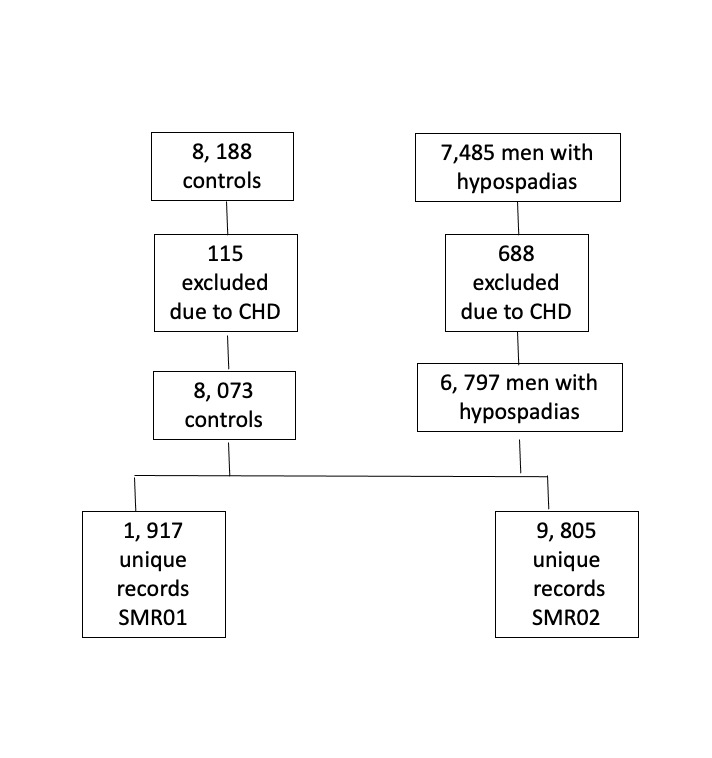


Supplementary Figure 8. Study selection data for data linkage studies. Abbreviations: SMR01: Scottish Morbidity Record 01 (Inpatients); SMR02: Scottish Morbidity Record 02 (Maternity).

**REFERENCES**

1. Lucas-Herald AK, Kyriakou A, Alimussina M, et al. Serum Anti-Müllerian Hormone In The Prediction Of Response To hCG Stimulation In Children With DSD. The Journal of Clinical Endocrinology & Metabolism. 2020.

2. Lurbe E, Agabiti-Rosei E, Cruickshank JK, et al. 2016 European Society of Hypertension guidelines for the management of high blood pressure in children and adolescents. J Hypertens. 2016;34(10):1887-920.

3. Flynn JT, Kaelber DC, Baker-Smith CM, et al. Clinical practice guideline for screening and management of high blood pressure in children and adolescents. Pediatrics. 2017;140(3):e20171904.

4. Obrycki Ł, Feber J, Derezinski T, et al. Hemodynamic patterns and target organ damage in adolescents with ambulatory prehypertension. Hypertension. 2020(119):14149.

5. Doyon A, Kracht D, Bayazit AK, et al. Carotid artery intima-media thickness and distensibility in children and adolescents: reference values and role of body dimensions. Hypertension. 2013(113):01297.

6. Maruhashi T, Soga J, Fujimura N, et al. Endothelial function is impaired in patients receiving antihypertensive drug treatment regardless of blood pressure level: FMD-J study (Flow-Mediated Dilation Japan). Hypertension. 2017;70(4):790-7.

7. Butlin M, Qasem A. Large artery stiffness assessment using SphygmoCor technology. Pulse. 2016;4(4):180-92.

8. Reusz GS, Cseprekal O, Temmar M, et al. Reference values of pulse wave velocity in healthy children and teenagers. Hypertension. 2010;56(2):217-24.

9. Shoji T, Nakagomi A, Okada S, Ohno Y, Kobayashi Y. Invasive validation of a novel brachial cuff-based oscillometric device (SphygmoCor XCEL) for measuring central blood pressure. J Hypertens. 2017;35(1):69-75.

10. Livak KJ, Schmittgen TD. Analysis of relative gene expression data using real-time quantitative PCR and the 2(-Delta Delta C(T)) Method. Methods. 2001;25(4):402-8.

11. Kinoshita H. Effects of oxidative stress on vascular function, and the role of anesthetics. J Anesth. 2012;26(1):141-2.

12. Akata T, Warltier DC. General anesthetics and vascular smooth muscle: direct actions of general anesthetics on cellular mechanisms regulating vascular tone. The Journal of the American Society of Anesthesiologists. 2007;106(2):365-91.
